# Three-dimensional morphology and gene expression in the *Drosophila *blastoderm at cellular resolution II: dynamics

**DOI:** 10.1186/gb-2006-7-12-r124

**Published:** 2006-12-21

**Authors:** Soile VE Keränen, Charless C Fowlkes, Cris L Luengo Hendriks, Damir Sudar, David W Knowles, Jitendra Malik, Mark D Biggin

**Affiliations:** 1 Berkeley Drosophila Transcription Network Project, Genomics Division, Lawrence Berkeley National Laboratory, One Cyclotron Road, Berkeley, California 94720, USA; 2 Berkeley Drosophila Transcription Network Project, Department of Electrical Engineering and Computer Science, University of California, Berkeley, California 94720, USA; 3 Berkeley Drosophila Transcription Network Project, Life Sciences Division, Lawrence Berkeley National Laboratory, One Cyclotron Road, Berkeley, California 94720, USA

## Abstract

**Background:**

To accurately describe gene expression and computationally model animal transcriptional networks, it is essential to determine the changing locations of cells in developing embryos.

**Results:**

Using automated image analysis methods, we provide the first quantitative description of temporal changes in morphology and gene expression at cellular resolution in whole embryos, using the *Drosophila *blastoderm as a model. Analyses based on both fixed and live embryos reveal complex, previously undetected three-dimensional changes in nuclear density patterns caused by nuclear movements prior to gastrulation. Gene expression patterns move, in part, with these changes in morphology, but additional spatial shifts in expression patterns are also seen, supporting a previously proposed model of pattern dynamics based on the induction and inhibition of gene expression. We show that mutations that disrupt either the anterior/posterior (a/p) or the dorsal/ventral (d/v) transcriptional cascades alter morphology and gene expression along both the a/p and d/v axes in a way suggesting that these two patterning systems interact via both transcriptional and morphological mechanisms.

**Conclusion:**

Our work establishes a new strategy for measuring temporal changes in the locations of cells and gene expression patterns that uses fixed cell material and computational modeling. It also provides a coordinate framework for the blastoderm embryo that will allow increasingly accurate spatio-temporal modeling of both the transcriptional control network and morphogenesis.

## Abstract 2

A new spatio-temporal coordinate framework for studying three-dimensional patterns of gene expression in the *Drosophila *blastoderm is presented that takes account of previously undetected morphological movements.

## Background

The transcription network controlling pattern formation in the *Drosophila *blastoderm is one of the best characterized animal regulatory networks [1-4] and, because of its relative simplicity, is one of the most tractable for computational modeling (for example, [5-8]). In this network, a hierarchical cascade of transcription factors drives expression of increasing numbers of genes in more and more spatially refined patterns through developmental stage 5. For example, along the a/p axis, the gap genes are among the first zygotically expressed transcriptional regulators, which cross-regulate each other and pair rule gene expression.

As part of the Berkeley *Drosophila *Transcription Network Project (BDTNP) [9], we have developed methods that convert images of whole blastoderm embryos into numerical tables describing the three-dimensional location of each nucleus and the relative concentrations of gene products proximal to each nucleus [10-13]. To utilize such data for modeling how the regulatory network generates spatial patterns of expression, it is critical to include temporal analysis because gene expression patterns change rapidly over time (for example, [2,7,14,15]). Since gene expression depends on the regulatory interactions between genes, these changes in patterns should give information on the structure of the network.

Two published observations suggest challenges to temporal modeling of the pregastrula regulatory network. First, the locations of gap gene stripes shift along the anterior/posterior (a/p) axis during stage 5 [6,7,15]. It has been proposed that this shift is caused by inductive and repressive interactions between the gap genes changing the extent to which cells express each gene. For example, a cell that, at an early time point, expresses a gap gene at the highest levels will later express this gene at lower levels, and neighboring cells to the anterior will now express this gene more strongly, resulting in an apparent motion of the expression pattern, a model we term 'expression flow'. Second, during nuclear division cycles 10 to 14, the local densities of nuclei change markedly along the a/p axis [16]. These density changes create the following difficulty. To model the network, it is necessary to compare changing gene expression profiles in embryos of different ages. Image analysis methods, however, only report the spatial location of nuclei, cells or expression features, and if their spatial coordinates change over time, it will be impossible to determine the correspondence between cellular expression in embryos of different ages unless we map how cells move. Indeed, if cells do change locations, then the measured shifts in expression stripe location reported by Jaeger *et al*. [7] may not in fact be due to expression flow. To identify the relative contributions of cell movement and expression flow to pattern dynamics, therefore, it is necessary to have a cellular resolution description of morphology at different developmental time points, along with an indication of corresponding nuclei across these time points.

To address this challenge, we have used our three-dimensional descriptions of blastoderm morphology and gene expression (described in [12]) to model the relative positions of nuclei at different time points during stage 5 and have compared these to changes in gene expression patterns. To test our model, we have also mapped cell movement in living Histone-green fluorescent protein (GFP) embryos. Our results show that nuclei and gene expression stripes move in previously uncharacterized, complex, three-dimensional patterns prior to gastrulation and that both morphological movements (which we term 'nuclear flow') and expression flow are together responsible for temporal changes in the spatial locations of gene expression patterns within the developing embryo.

## Results

### Complex changes in nuclear density patterns

**Figure 1:**
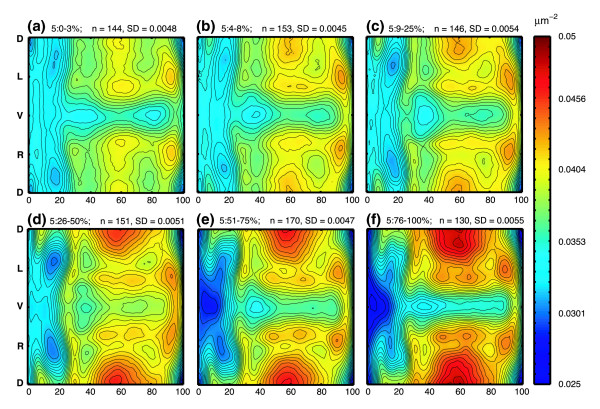
Changing local nuclear density patterns during stage 5. Average local nuclear densities on the blastoderm surface were computed for PointCloud data derived from embryos for six consecutive time intervals spanning stage 5. **(a-f) **Cylindrical projections of the average for each of these temporal cohorts. The range of membrane invagination for embryos in each temporal cohort is shown above each panel (for example, 5:0-3%). Isodensity contours were plotted over a color map representing local average densities from 0.025 nuclei/μm^2 ^(dark blue) to 0.05 nuclei/μm^2 ^(dark red). The position on the y-axis of the dorsal midline (D), ventral midline (V), and left (L) and right (R) lateral midlines are indicated. On the x-axis, anterior is to the left, posterior is to the right, and the distance along the a/p axis is given as a percent egg length. The number of embryos in each cohort (n) and the standard deviation of nuclear density values (SD) are also shown. It can be seen that over time the local nuclear densities increased dorsally, decreased at the poles, and changed little ventrally.

The accompanying paper established that a temporal cohort of late stage 5 embryos has a complex and highly reproducible three-dimensional pattern of nuclear densities [12]. The work presented here shows that nuclear density patterns also change dramatically during stage 5 (Figure 1). In early stage 5 embryos, several patches of high nuclear density were seen, including two lateral, two posterior and one dorsal patch. As stage 5 progressed, nuclear densities decreased at the poles of the embryo, especially anteriorly; densities increased dorsally in the middle of the embryo; and densities remained largely unchanged ventrally in the middle of the embryo.

The observed increases in nuclear density dorsally could not have been caused by localized division of nuclei since the nuclei/cells do not divide during stage 5, nor is there evidence that nuclei are preferentially destroyed at the poles of the embryo [17,18]. This was further confirmed by our data, as the total number of nuclei detected per embryo remained the same for most of stage 5 (Table 1). Therefore, the local changes in density must have resulted from movement of nuclei either towards each other, where densities increased, or apart from each other, where densities decreased.

The previous temporal analysis only examined changes in nuclear density between consecutive nuclear division cycles and, thus, could not rule out preferential nuclear division or loss as a major cause of nuclear density differences [16]. Our data establish that, during stage 5, morphological movements are responsible for the density changes observed. This is surprising as these movements occur well before gastrulation at a time when cells were previously not thought to move [19].

### Nuclear density patterns and movements in living embryos

The observed changes in nuclear density might have been caused by some artifact in embryo preparation, such as preferential shrinkage or expansion of different regions of the embryo during fixation, mounting and so on. To verify the accuracy of our nuclear density maps based on fixed material, we used embryos expressing Histone2A-GFP to measure both nuclear density and the movement of individual nuclei over the course of stage 5 in living embryos [16,20]. Images of each embryo were recorded every few minutes and the resulting time-lapse image series were used to track individual nuclei automatically through stage 5.

A technical limitation of our live embryo studies was that a patch of only about 20% of each embryo could be imaged because of lower signal to noise and the higher light scatter associated with living cells. Consequently, we imaged patches of 22 embryos that were in different orientations and combined these data to provide an overview for much of the surface of the embryo. Our live embryo data do not have as high a resolution as the data derived from fixed material because living embryos moved slightly during imaging, mapping patches of two-dimensional data from multiple embryos on to a common frame was imprecise, and our sample size was smaller.

**Figure 2:**
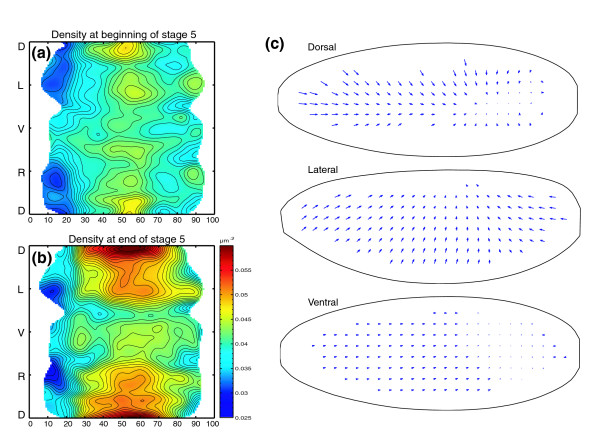
Nuclear density patterns and movements in living Histone2A-GFP embryos. **(a,b) **Cylindrical projections of average nuclear density maps derived from portions of 22 living embryos expressing Histone2A-GFP. Density maps for this set of embryos are shown at the start of stage 5 (a) and at the end of stage 5 (b). The axis and isodensity contours are labeled as in Figure 1. The density patterns seen are remarkably similar to those derived from fixed material (Figure 1). **(c) **Orthographic projections of the average distance and direction of nuclear movement in two dimensions for *n *= 1 embryo in the dorsal orientation, *n *= 8 embryos in the lateral orientation, and *n *= 4 embryos in the ventral orientation. These arrows represent a local average movement within each embryo calculated from time-lapse series as well as an averaging over the various embryos in similar orientations. As expected from the changes in nuclear densities, a net flow of nuclei from the poles towards the mid-dorsal region was observed.

Despite these limitations, the nuclear density patterns seen in the live embryos at the beginning and end of stage 5 broadly resembled those seen in the fixed material (compare Figures 2a and 1a, and compare Figures 2b and 1f). In addition, the live data confirm that nuclei move qualitatively in the manner predicted by the density changes seen in the fixed material. Nuclei flowed from the anterior and posterior towards the middle of the embryo; this movement was greater dorsally than ventrally; and there was a tendency for nuclei to move from ventral to dorsal in the center of the embryo (Figure 2c; Additional data file 1). Hence, the live embryo data show that the observed three-dimensional changes in density patterns are not an artifact of fixed embryo preparation. Further, the measured nuclear movement is significant as it was as large as 20 μm, or 3 cell diameters, motivating the need to model these movements.

### Modeling nuclear movements from fixed embryo data

To model temporal changes in gene expression patterns in blastoderm embryos, it is critical to know which nuclei/cells are equivalent in embryos of different developmental stages. The analysis of nuclear movements in live embryos did provide such correspondences for a limited number of nuclei in individual embryos (Figure 2c), but these data are neither accurate enough nor comprehensive enough to be used to predict nuclear correspondences between entire PointClouds. Instead, we used whole embryo PointClouds from multiple temporal cohorts to build a numerical model that predicts the direction and distance that each nucleus needs to move through space in order to account for the measured changes in nuclear density.

Based on the behavior of nuclei observed in our live embryo studies, our model assumed that the total number of nuclei does not change, that the flow of nuclei was smooth, and that the total flow movement was small. Because the average shape and surface area of PointClouds changed during stage 5 (see below) these data were also included in our model. A synthetic embryo was constructed by placing 6,078 nuclei in order to optimally match the average shape and nuclear density pattern measured in the earliest stage 5 cohort. Then, nuclei were allowed to flow, respecting the above constraints, to obtain a density pattern and shape that most closely matched that of the latest stage 5 cohort. The resulting flow provided the needed correspondence between early and late stage 5 nuclei.

**Figure 3:**
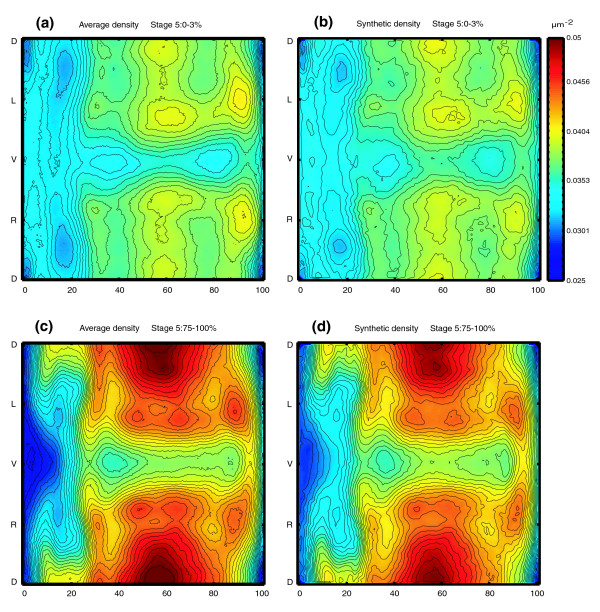
Synthetic density maps are similar to measured density maps. Cylindrical projections of nuclear density patterns in PointClouds from: **(a) **fixed early embryos (stage 5:0-3%); **(b) **an early synthetic embryo modeled to have shapes and nuclear density patterns of stage 5:0-3% fixed embryos; **(c) **fixed late embryos (stage 5:75-100%); and **(d) **a late synthetic embryo modeled to have shapes and density patterns of stage 5:75-100% fixed embryos according to the model described in the text. All other information and scales are as used in Figure 1.

**Figure 4:**
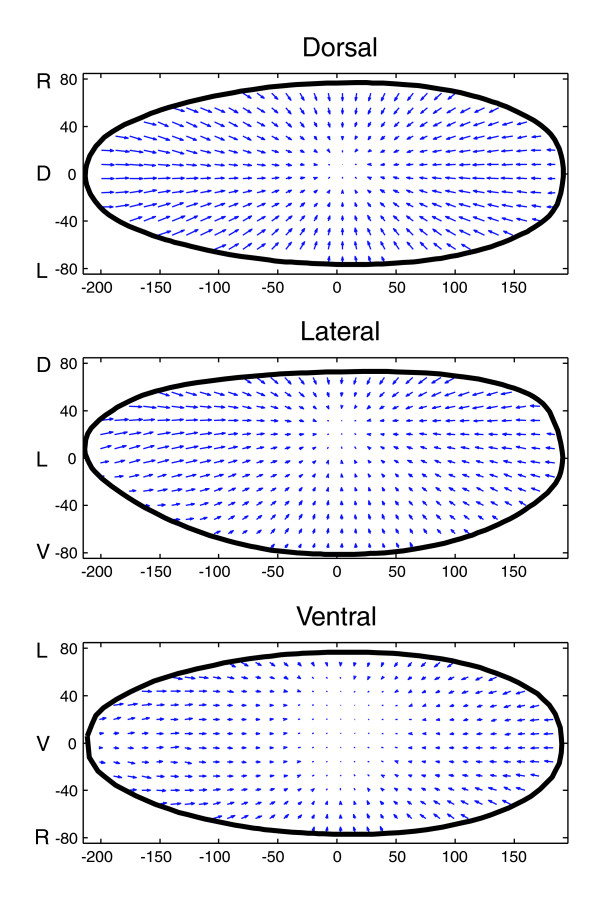
Predicted nuclear flow in the stage 5 blastoderm. The movement of nuclei in three dimensions was estimated using PointCloud data derived from fixed embryo material. Three orthographic projections of this model are shown, illustrating movement dorsally (top), laterally (center), and ventrally (bottom). The length and direction of arrows indicate the direction and distance of nuclear movement. The position on the y-axis of the dorsal midline (D), ventral midline (V), and left (L) and right (R) lateral midlines are indicated. On the x-axis, anterior is to the left and posterior to the right. The scale is in μm from the embryo center of mass. The predicted movements broadly agree with those seen in the live embryos, being greater at the poles and dorsally than ventrally. Note that the apparent movement towards the center of each view results from the basal movement of nuclei inward.

The synthetic nuclear density maps produced agreed closely with maps measured from actual embryos at the same stages of development (Figure 3). Although density alone was a fairly weak constraint, the model's requirement of a small, smooth movement resulted in a solution that was quite robust to perturbations of the constraints and initial conditions. Figure 4 shows the map of predicted nuclear movements between the early and late synthetic embryos. Qualitatively, the predicted movements matched those observed in the live data, showing larger movements at the poles and dorsally than ventrally. Quantitatively, the movements were of a similar order (compare Figures 4 and 2c). For example, the flow along the lateral midline 150 μm posterior of the embryo's center of mass was 5 μm in the live data and 6 μm in the model. We also tested a variant of our model for nuclear movements in which the density data from all six temporal cohorts were used. This yielded a nearly identical pattern of overall movement, further validating the assumption of slow, smooth motions.

**Figure 5:**
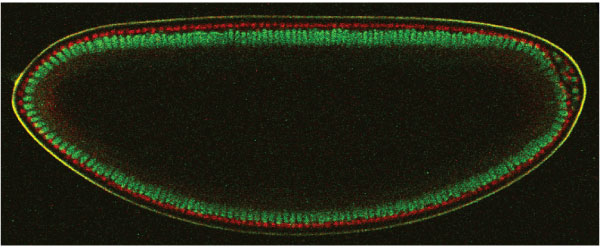
Basal movement of nuclei during stage 5 in living Histone2A-GFP embryos. Two optical slices through the middle of a living embryo are superimposed. The red image was taken at the beginning of stage 5, whereas the green image was taken at the end. The bright line is the water-oil interface at the vitelline membrane, and was used to align the two images. All nuclei move inwards and elongate during stage 5. Anterior is to the left and dorsal is up.

The movements predicted by our model also showed that the nuclear centers of mass move inwards, that is, basally, towards the center of the embryo. This basal movement is visible in all three orthographic views of the predicted movements shown in Figure 4 as a flow inwards, towards the center point of each projection. Although this was not apparent from the two-dimensional data taken on the surface of Histone2A-GFP embryos, an optical cross-section taken of all 22 live embryos confirmed the same uniform basal nuclear movement of about 5 to 8 μm around the entire blastoderm surface (for example, Figure 5). Thus, there are at least two components to the nuclear movements in the blastoderm: a basal movement that alone would cause nuclear densities to increase everywhere, and a flow of nuclei parallel to the surface that causes differential density decreases and increases in specific regions.

### Temporal changes in gene expression patterns

**Figure 6:**
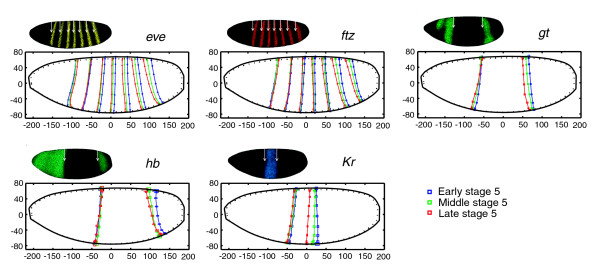
Movement of gap and pair rule stripe borders. Lateral orthographic projections of the mean positions of the anterior borders of *eve *and *ftz *stripes from early (4-8%) (blue), mid (26-50%) (green) and late (76-100%) (red) stage 5 cohorts, and of selected borders of *gt*, *hb *and *Kr *stripes from early (0-3%) (blue), mid (9-25%) (green), and late (51-75%) (red) stage 5 cohorts. The stages chosen for gap gene analysis were earlier than those for pair rule genes because, unlike pair rule mRNA, gap mRNA is rapidly down-regulated towards the end of stage 5, whereas pair rule expression increases throughout stage 5. The error boxes at each measurement point represent 95% confidence intervals for the mean in a/p and d/v directions. Anterior is to the left, dorsal is to the top. The x- and y-axes show the distance in μm from the center of embryo mass. It can be seen that most stripe borders changed spatial location during stage 5. The silhouettes of PointClouds were smaller for later stage embryos because of basal nuclear movements. Note that, for each *eve *and *ftz *stripe, the posterior stripe border shows a broadly similar movement to the anterior border, indicating that the movements observed are not principally due to the narrowing of stripes (data not shown).

Having established a model for nuclear movement during the course of stage 5, we then measured how the borders of expression stripes of several gap and pair rule genes shifted over this same time as a step towards determining the relationship between nuclear movement and changes in gene expression patterns. We mapped the average positions along the a/p axis of selected borders of expression stripes for the gap genes *hunchback *(*hb*), *Krüppel *(*Kr*), and *giant *(*gt*) and for the pair rule genes *even-skipped *(*eve*) and *fushi tarazu *(*ftz*). PointClouds from each temporal cohort were aligned and scaled to the mean a/p axis length of the cohort, and the locations of stripe borders were calculated for each of 16 strips around the circumference of the embryo (see [12]). Figure 6 shows lateral orthographic projections of these data for the nine strips on one side of the embryo.

As described in the introduction, previous one-dimensional analyses showed that some stripes of gap expression move along the a/p axis during stage 5, and it was proposed that these movements resulted from cross-regulatory interactions among the gap genes causing an expression flow across the field of cells [7]. Our three-dimensional data are consistent with the published observations on stripe movement but, in addition, they show that the degree to which stripes shifted location along the a/p axis differed considerably at different points around the circumference of the embryo (Figure 6). For example, between the earliest stage 5 cohort and the oldest stage 5 cohort, the more posterior border of *hb *expression shown in Figure 6 moved 2.4 times further along the a/p axis on the dorsal midline than it did on the ventral midline (26 μm versus 11 μm).

Another striking feature of our data was that the stripe borders moved differently from each other in the same region of the embryo. For example, *eve *stripe borders moved to a greater extent than did adjacent *ftz *stripes (for example, the posterior edge of *eve *stripe 7 moved 15 μm ventrally whereas the posterior edge of *ftz *stripe 7 moved 6 μm ventrally); and the posterior border of the *Kr *stripe moved much more than the nearby *ftz *stripe 4, especially ventrally, where the movement was 7 times larger (Figure 6). The temporal dynamics of movement were also different for each gene: for example, the posterior *hb *stripe border moved most in early stage 5, whereas the *Kr *posterior border moved more at later times (compare *hb *and *Kr *in Figure 6). Thus, the nuclear motions we observed cannot account for all of the changes in stripe locations as morphological movements would have affected all stripes equally at the same place and time. To this extent, our data immediately support the expression flow model: changes in the spatial location of at least some gene expression features must have resulted from changing relative levels of expression within given cells.

### The relative contributions of nuclear flow and expression flow to pattern flow

Since nuclear movement must also play a role in driving the changes in stripe location observed, we next sought to determine the relative contribution of both nuclear movements and expression flow to the stripe movement. To do this, we used our model of nuclear movements (Figure 4) to predict for each stripe how far and in what direction it would be expected to move due to nuclear movement alone, a distance we term 'nuclear flow'. We then compared this nuclear flow to the total distance that the stripe border moved, a distance we term 'pattern flow'. The part of pattern flow not explained by nuclear flow should be due to expression flow. In other words, pattern flow = nuclear flow + expression flow.

**Figure 7:**
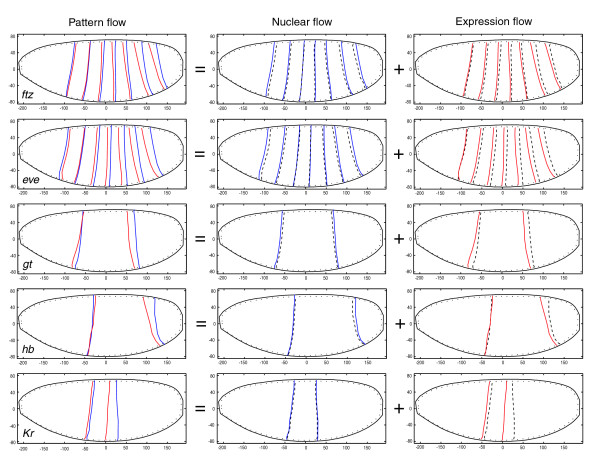
The relative contributions of nuclear flow and expression flow to pattern flow. Orthographic projections of the locations of *ftz*, *hb, eve*, *gt*, and *Kr *stripe borders in early stage 5 (blue lines) and late stage 5 (red lines) embryos. The stripe locations are taken from the earliest and latest applicable embryo cohort (5:4-8% to 5:75-100% for *ftz *and eve; 5:0-3% to 5:51-75% for *hb*, *gt and Kr*). The axes are labeled as in Figure 4. Our model of nuclear flow was used to predict the location of stripe borders in late embryos in the absence of changing expression levels (dotted black lines). The left panels compare the measured locations of the early and late stripe borders, and thus show the pattern flow. The center panels show the movement predicted to be due only to nuclear flow. The right panels show the residual movement (expression flow) that can be attributed to zones of up/down-regulation along stripe boundaries.

The results of this analysis are shown for *ftz*, *eve*, *Kr*, *gt *and *hb *in Figure 7. It can be seen that the degree of nuclear flow and expression flow were generally of a similar order and thus both were significant in determining the extent of pattern flow. Interestingly, expression flow always moved stripe border locations from posterior to anterior over time, whereas cell flow moved stripes towards the middle of the embryo along the a/p axis. Thus, in the anterior of the embryo the two mechanisms tend to counteract each other, while in the posterior they reinforce each other.

### A morphological interaction between the anterior/posterior and dorsal/ventral networks

It seems reasonable that expression flow results from the cross-regulatory interactions between gap genes as proposed [7]. But what regulates nuclear flow? Blankenship and Wieschaus showed that nuclear density along the a/p axis is regulated by *bicoid *(*bcd*) [16], a primary maternal determinant of a/p patterning [21,22]. Similarly, it seems probable that the primary maternal determinants of dorsal/ventral (d/v) patterning regulate densities along the d/v axis. As our three-dimensional data show, however, d/v and a/p morphology are strongly coupled by the geometry of the blastoderm. As nuclei move in three dimensions, they change in both the a/p and d/v coordinates simultaneously. Therefore, it is likely that genes controlling density patterns along one axis would also affect density patterns, nuclear flow, and thus pattern flow along the other axis.

Interactions between the a/p and d/v regulatory systems are rarely considered, but subtle effects of the d/v system on pair rule stripe patterns have been noted [23-25], which these authors proposed resulted from direct induction or repression of a/p system components by d/v transcriptional regulators. Since our data suggest an alternative possibility, we tested the role of both the a/p and d/v networks in controlling nuclear densities and pattern flow in order to measure any interaction between the a/p and d/v regulatory systems and see if this could be explained, at least in part, via the effects of morphological movement.

We mapped nuclear density patterns in embryos mutant for either *bcd *or one of two d/v patterning genes, *gastrulation defective *(*gd*) and *Toll *(*Tl*). We also measured changes in the positions of *ftz *stripes along the a/p axis in *gd *and *Tl *mutants. In embryos lacking *gd*, the whole blastoderm takes on a dorsal fate [26,27], whereas in dominant active *Tl *mutants the whole blastoderm is ventralized [28].

**Figure 8:**
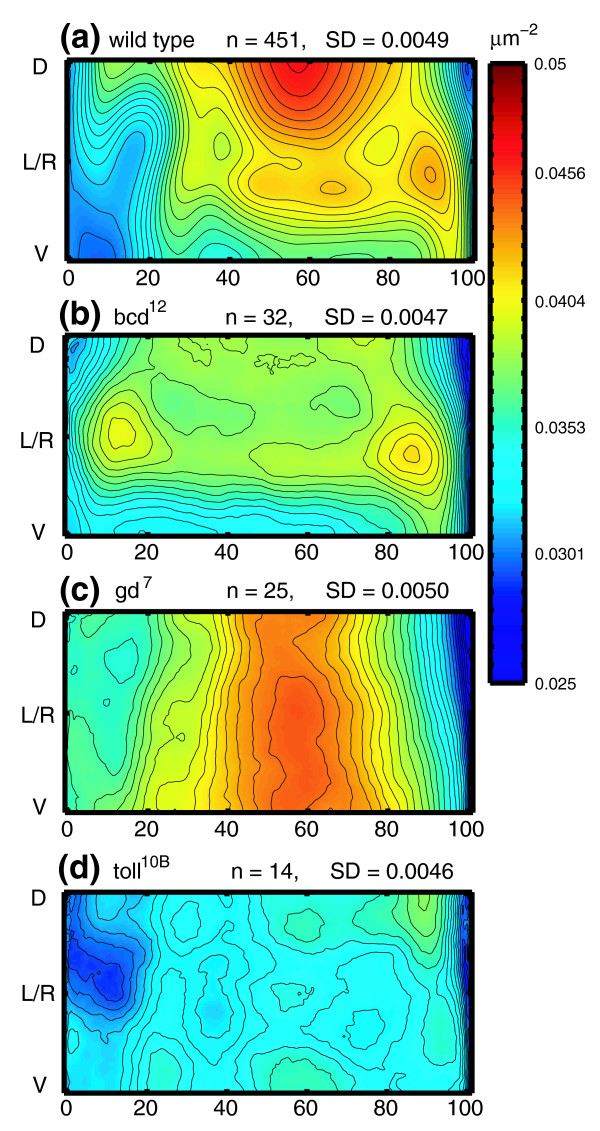
*bcd*, *gd*, and *Tl *regulate nuclear density patterns along both major body axes. Cylindrical projections of nuclear density patterns in **(a) **wild-type, **(b) ***bcd*^12 ^mutant, **(c) ***gd*^7 ^mutant, and **(d) ***Tl*^10*B *^mutant embryos. To reduce noise, information from the left and right sides of each embryo was averaged. All embryos were from stages 5:25-100%. Axes and isodensity contours are as described in Figure 1. All three mutants exhibit changes in the pattern of density along both body axes. Note that while it appears that the total number of nuclei in *Tl*^10*B *^mutants is less than in the wild-type embryos, this reflects a difference between fly strains and not an effect of the *Tl *gene as there is no statistically significant difference between the average number of nuclei in *Tl*^10*B *^mutants versus their wild-type-like siblings, which are derived from *Tl*^10*B *^hetrozygous mothers.

Figure 8 shows that *gd *and *Tl *both regulate density patterning along the d/v axis and, as shown previously, *bcd *regulates patterning along the a/p axis. The density map for *gd *mutants most resembled the pattern seen along the dorsal midline in wild-type embryos, and the map for *Tl *mutants most resembled that seen along the ventral midline in wild-type embryos, consistent with these two genes' roles in d/v patterning. Strikingly, however, mutations in *bcd*, *gd *and *Tl *also affected the density map along the alternative body axis. For example, in embryos lacking functional *Bcd*, the patch of high nuclear density that developed dorsally in wild-type embryos during stage 5 was greatly reduced, dramatically altering density patterns along the d/v axis. Similarly, in *gd *mutant embryos, the a/p profile differed significantly from that along the dorsal midline of the wild type, with a lower peak of density. In addition, a/p patterning features, such as the ridge of high density that corresponds to the precephalic furrow region [12], were largely absent. Thus, the a/p and d/v regulatory networks do interact, at least in part, via their control of nuclear movements.

**Figure 9:**
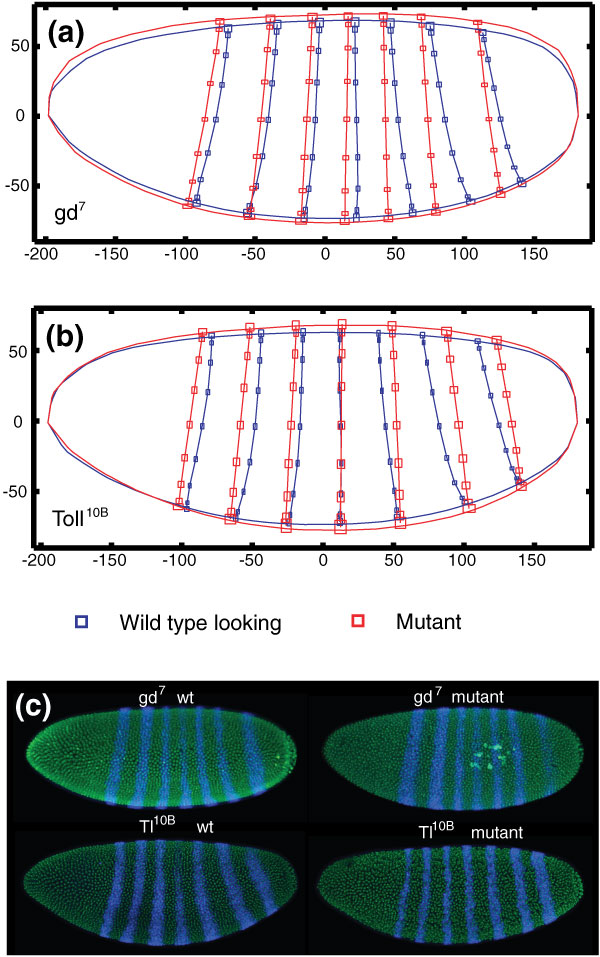
*gd *and *Tl *regulate *ftz *stripe location. Quantitative comparison of *ftz *expression **(a) **between mutant embryos derived from *gd*^7 ^homozygous mothers and wild-type-like embryos derived from *gd*^7 ^heterozygous mothers and **(b) **between mutant embryos derived from *Tl*^10*B *^homozygous mothers and wild-type-like embryos derived from *Tl*^10*B *^heterozygous mothers; both show lateral orthographic projections indicating the position of each of the seven stripes in wild-type-like embryos (blue stripes) and mutant embryos (red stripes). All embryos were from stages 5:25-75%. The confidence intervals, embryo orientation, and scales are as described in Figure 4. Shifts in the *ftz *expression boundaries are consistent with dorsalized (*gd*) and ventralized (*Tl*) nuclear flow, respectively. **(c) **The effects of disrupting the d/v system on stripe curvature and placement in single embryo images, shown in a lateral view. The stripes in the mutant embryos (right) clearly differ from those in the wild-type-like embryos (left), but because of small differences in embryo orientation and shape it is difficult to draw a precise understanding of how stripe locations have changed from such raw image data.

We have not modeled the nuclear movements in these mutants but, given the nuclear density patterns, the nuclear flow in the a/p direction will be much more similar dorsally and ventrally in *gd *and *Tl *mutant embryos than in wild-type embryos. Figure 9 shows that, in *gd *and *Tl *mutant embryos, the locations of *ftz *stripes were shifted in a way consistent with this prediction. In ventralized *Tl *mutant embryos, the *ftz *stripes were located normally ventrally (that is, located as they are in wild-type-like embryos), but were spaced further apart dorsally than in wild-type-like embryos, consistent with the reduced nuclear flow expected in this mutant. In dorsalized *gd *embryos, the opposite result was observed: the spacing of *ftz *stripes was only affected in the ventral region, where they were closer together than in wild-type-like embryos. Strikingly, in both *Tl *and *gd *mutants all of the *ftz *stripes were straight, whereas in wild-type embryos pair rule and gap gene stripes have a distinct curve [12] (Figures 6 and 9).

### A transcriptional interaction between the a/p and d/v networks

**Figure 10:**
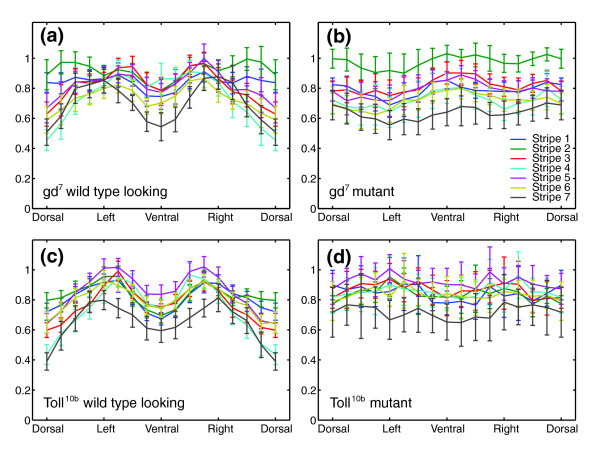
*gd *and *Tl *regulate the levels of *ftz *stripe expression in the direction of the d/v axis. Plotted are averaged expression intensities of gene stripes for *ftz *in wild-type-like embryos derived from **(a) ***gd*^7 ^heterozygous mothers, **(b) **dorsalized mutant embryos from *gd*^7 ^homozygous mothers, **(c) **wild-type-like embryos from *Tl*^10*B *^heterozygous mothers, and **(d) **ventralized mutant embryos derived from *Tl*^10*B *^homozygous mothers. Expression intensity (y-axis) is plotted against the location along the stripe in the direction of the d/v axis as described in [12]. Each of the seven *ftz *stripes is shown in a different color and the error bars give the 95% confidence intervals for the means. The differences in expression seen in wild-type embryos in the direction of the d/v axis are not seen in the mutant embryos.

As explained in more detail in the Discussion, the effect of the d/v network on pair rule gene stripe location could be explained entirely by the d/v system's control of cell movements. In the accompanying paper [12], however, we showed that there are quantitative changes in the levels of pair rule expression along the direction of the d/v axis. It is difficult to imagine how these could be caused by such an indirect morphological effect. Instead, such changes in expression levels are likely to be caused by transcriptional control of either the pair rule genes or their gap gene regulators by the d/v system. To verify that these quantitative changes in pair rule expression levels are controlled by the d/v network, we compared expression of each of the seven *ftz *stripes in wild-type-like and *Tl *and *gd *mutants. As Figure 10 shows, the modulations in expression levels in the direction of the d/v axis in wild-type embryos are no longer seen in either mutant background, suggesting that the interaction between the d/v and a/p networks in the pregastrula embryo likely includes morphological and transcriptional mechanisms.

## Discussion

### A new coordinate frame for modeling the blastoderm network

The BDTNP is developing methods to measure and model gene expression in stage 5 *Drosophila *embryos [10-13]. Previous models of the pregastrula network assumed that, during stage 5, cells and nuclei do not move and that those gene expression features that change their spatial locations must be moving across a field of static cells [6,7]. Here we have established that, over the course of stage 5, there are spatial translocations of nuclei of up to 20 μm, or about 3 cell diameters. These movements are large relative to the system's precision in specifying spatial patterns, which can change dramatically even between neighboring cells. Therefore, to allow more accurate analysis of the regulatory network, we have built a model of the three-dimensional movement for all of the approximately 6,000 nuclei in the cellular blastoderm. This ensures that expression at the same cellular locations can be studied at different time points. In effect, we have created a spatio-temporal coordinate system based on equivalent cells, while previous analyses of the blastoderm have relied on coordinate systems based on absolute spatial location alone.

### A novel strategy for following cells/nuclei during development

Establishing a suitable coordinate system is a general problem in developmental biology because cells move dynamically. At times these movements are obvious to the human eye, such as the rapid movements of gastrulation. There are many examples, however, of tissues like the *Drosophila *cellular blastoderm that appear, by eye, to be relatively static. We suspect that many of these tissues will also be found to be dynamic when their morphology is quantified at cellular resolution by methods such as those we have employed.

Developmental biologists typically follow the locations of cells over time using live cell imaging, but this approach is often limited by poor signal to noise and high light attenuation along the z-axis of the microscope. Imaging fixed material can overcome these difficulties as it is generally possible to achieve higher quality fluorescent staining and, by selecting proper embedding media, to increase transparency of the tissue. Our computational model for predicting nuclear/cellular movements relies on having enough data to estimate the average positions of all cells in the embryo or tissue under study. We suggest that, when it is possible to obtain such data for cohorts at different time points, the strategy we have employed based on fixed material provides a powerful alternative to live cell analysis for quantifying nuclear/cell movements.

### Distinguishing expression flow and nuclear/cellular flow

Our work has provided critical verification of the expression flow model of Jaeger *et al*. [6,7]. Because we have been able to estimate what fraction of the movement of gene expression stripes is due to morphological movement, we have been able to rule out the possibility that all of the measured stripe movements are due to morphological change and have been able to provide a more precise estimate of the rate and degree of expression flow across the embryo.

### Temporal changes in stripe location are not one-dimensional

The original model that suggested expression flow in the embryo was based on one-dimensional descriptions of gap gene expression along the a/p axis [6,7]. Our three-dimensional data show clear differences between dorsal and ventral regions in the degree of expression flow for pair rule and gap gene stripes along the a/p axis as well as temporal differences between genes (Figure 7). Thus, actual gene expression patterns are far more complex than current models indicate. Models of pattern formation that can account for this complexity will be better justified and may well uncover novel regulatory mechanisms not detectable in one-dimensional data.

### Interactions between d/v and a/p pattern formation

The early a/p and d/v regulatory cascades are generally described as acting independently of one another [2,29,30]. Several groups, however, have noted subtle effects of the d/v patterning system on pair rule gene expression, including *ftz*'s [23-25]. Our quantitative analysis extends these previous observations and, in some cases, suggests alternative models.

Carrol *et al*. [24] noted that the d/v system regulates the spatial locations of the *ftz *stripe in a way qualitatively consistent with the changes we observed (Figure 9). It was proposed that stripe locations varied along the d/v axis due to d/v regulators directly or indirectly affecting ftz transcription [24]. As in the expression flow model, it was assumed that the field of cells in the embryo is static and that disruption of the d/v system changed in which cells *ftz *is maximally expressed. Our nuclear density data suggest an alternative mechanism: the changes in *ftz *stripe location in embryos in which the d/v system had been disrupted (Figure 9) are consistent with an indirect effect of the d/v system on *ftz *stripe location via d/v control of nuclear movements (Figure 8). In wild-type embryos, nuclei dorsally move further from the poles towards the middle of the embryo than they do ventrally (Figures 2 and 4), whereas in d/v mutant embryos the density patterns suggest this d/v difference in movement does not occur (Figure 8). *ftz *stripe locations in d/v mutant embryos are shifted, compared to those in wild-type embryos, as would be expected from the effect of d/v genes on nuclear movements (Figure 9). Thus, part and perhaps all of the d/v system-induced shift in *ftz *stripe locations result from nuclear flow.

Interestingly, our data show that, when d/v patterning is disrupted, the normally curved *ftz *stripes become remarkably straight (Figure 9). It has previously been suggested that curving of pair rule stripes is caused by geometric constraints of the *Drosophila *egg on diffusion based a/p patterning signaling [31]. We suspect, however, that the curvature of pair rule stripes is not a result of the a/p patterning system - whatever mechanism(s) it employs - but instead results from the effect of the d/v system on nuclear/cell movements. Future attempts to compare the expectations of specific models with observed gene expression patterns will benefit greatly from accurate three-dimensional descriptions of gene expression and morphological dynamics at cellular resolution.

Changes in the levels of pair rule gene expression between dorsal and ventral regions have been noted previously in a few cases [23,25]. In most cases, the changes noted have been transient, being observed only during early stage 5, and have been sufficiently large to be detected by visual inspection of two-dimensional photographs. Our studies have established that, later during stage 5, quantitative differences in *ftz *expression are found along stripes in the direction of the d/v axis that are not readily apparent by visual inspection of stained embryos [12] (Figure 10). The late stage 5 patterns we have measured differ significantly from the transient patterns noted previously.

Clearly, the d/v systems' regulation of differential expression levels between dorsal, lateral and ventral locations along pair rule stripes cannot be due to nuclear flow. Instead, it must result from direct transcriptional control of at least some members of the a/p regulatory network by d/v factors. From the work presented here, we cannot determine which a/p genes are the direct targets of d/v regulators but separate experiments by the BDTNP have established that combinations of the d/v factors *dorsal*, *twist *and *snail *bind to many known pair rule gene regulatory enhancers, including ones in *ftz *(X Li, S MacArthur, R Bourgon, D Nix, HC Chu, M Eisen, M Biggin, personal communication). We suggest that the effects of the d/v system on a/p patterning genes is more pervasive and complex than previously appreciated and that it is likely that these effects are biologically significant.

## Materials and methods

### Fly stocks

The strains used to examine the effect of maternal mutations on embryo morphology were *bcd*^12^, p^p^/TM3, Sb^1 ^(#3444 Bloomington Stock Center, Bloomington, IN, USA); *gd*^7^/FM3 [32,33] (courtesy of M Levine); and *Toll*^10*B*^/TM3, Sb, Ser x *Toll*^10*B*^/or60 [34,35] (courtesy of M Levine, UC Berkeley, CA, USA). *bcd*^12 ^is a strong loss of function allele [22], *gd*^7 ^is an amorphic point mutation [27], and *Toll*^10*B *^is a constitutively active point mutation [28,36]. Analysis of nuclear densities and movement in live embryos were performed using transgenic Histone2A-GFP embryos [20] (courtesy of E Wieschaus, Princeton University, NJ, USA).

### Generation of PointClouds

The methods used to obtain PointCloud files and visualize them were as described in [12].

### Live embryo imaging

Live cell movements were recorded as time series from Histone2A-GFP embryos mounted in halocarbon oil 700 between a membrane and a coverslip [37]. The embryos were collected for 3 h and dechorionated for 2 minutes in 50% bleach. Three-dimensional images were obtained using two-photon excitation at 850 nm and 250 mW (at the source), and a 20 × 0.75 NA planapo lens. Each embryo was imaged every 2 to 5 minutes while it developed from late stage 4 to stage 6.

Because a living embryo is opaque and the imaging time per frame is limited, only the portion of the embryo closest to the coverslip was imaged (about 20% of the surface). To correct for embryo movement during imaging, an image slice was taken through the center of the embryo both at the beginning and at the end of stage 5. The angle of the d/v rotation of the embryo was estimated by observing the location of the ventral furrow or the movement of the pole cells after the end of stage 5. To eliminate embryos with abnormal morphogenesis caused, for example, by hypoxia, all embryos were allowed to develop until the following day. Only images from embryos where development appeared undisturbed up to stage 17 were used in subsequent analyses.

### Image analysis of live embryo data

All image processing and analysis algorithms used were implemented in MATLAB (The MathWorks Inc., Natick, MA, USA) with the DIPimage toolbox [38,39]. Each three-dimensional image was collapsed into a two-dimensional image by detecting the region containing the nuclei and performing a maximum-projection onto the imaging plane. Combining the result of a watershed [40,41] and an isodata threshold [42] produced a nuclear segmentation mask that was near-perfect in a large portion of the imaged surface. Due to the projection, the segmentation quality was poorer near the periphery where the embryo surface curves away from the microscope objective. Locations of nuclei were given by the center of mass of the fluorescence intensity within each segmented region.

**Figure 11:**
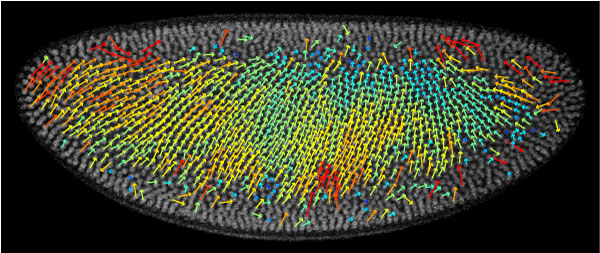
The distance and direction of nuclear movement in an individual living Histone2A-GFP embryo. Nuclei were tracked throughout stage 5, and the vector of their total motion was plotted on top of the image of the embryo at the beginning of stage 5. The color of the arrows is given by their length, short arrows being blue and long ones red. Vectors that are very different from nearby vectors were not used when generating the averaged plots in Figure 2. Anterior is to the left and dorsal is up.

Nuclear densities were computed by counting the number of nuclei in a 10 μm radius circle around each nucleus. Nuclei were tracked through the time lapse sequence by matching the neighborhood configuration of one nucleus near the center of the image and iteratively propagating the match to neighboring nuclei. The absolute distance was recorded for nuclei that could be tracked from the first image within stage 5 to the last (Figure 11). To correct for small rotations and translations of the embryo that might have occurred during the imaging process, the initial and final positions were aligned based on the location of the vitelline membrane using the optical slices described above, imaged through the middle of the embryo (Figure 5). Both the two-dimensional projections of the time series through stage 5, as well as the slices through the middle of the embryo at the beginning and end of this stage, for each of the 22 embryos used here, are provided in Additional data file 1.

Nuclear density data from multiple embryos were mapped onto a cylindrical projection using the center of mass and moments of inertia to align the a/p axis of embryos, scaling the coordinates to match the average cohort egg length for the PointClouds derived from fixed embryos, and transforming the image coordinates (*x*, *y*) into cylindrical coordinates (*x*, *φ*) using the width of the embryo (taken from the mid-embryo image) and the d/v orientation. The mapped data were then smoothed and resampled to a regular grid using normalized convolution [43]. Nuclear movement data were mapped to an orthographic projection using the same method.

### Temporal analysis of PointCloud data from fixed embryos

PointCloud files describing relative levels of gene expression per nucleus were generated [12]. PointCloud files were grouped into six temporal cohorts according to their stage. To register PointClouds for each temporal cohort, individual PointClouds were translated to align the center of mass and rotated to align the principal (a/p) axis with a standard coordinate system. To align PointClouds in the d/v orientation, the ventral midline was determined based on the expression pattern of a d/v gene (typically *snail*) or the d/v asymmetry present in an a/p marker (usually *ftz *or *eve*). After these rigid alignments, PointClouds within a cohort were subsequently scaled isotropically to match the egg length to the cohort average.

The a/p locations of boundaries of pair rule and gap gene expression were measured for 16 points around the embryo circumference for each temporal cohort [12].

Nuclear densities were calculated for each cohort essentially as in [12]. To reduce the effect of the nuclear segmentation errors on the sides of the image due to limited z-resolution, density averages were computed in a weighted manner. For each embryo *i*, and cylindrical coordinate (*x*, *φ*) a weight, *W*_*i*_(*x*, *φ*), was assigned as the cosine of the angle between the surface normal at (*x*, *φ*) and the z-axis of the image. The average density at a given spatial coordinate was then given by the weighted average:

D^ave^(*x*, *φ*) = Σ_i _W_i_(*x*, *φ*) D_i_(*x*, *φ*)/Σ_i _W_i_(*x*, *φ*)

A similar computation yielded spatially weighted variance and confidence estimates. This weighting scheme made the density estimates more robust for small cohorts but was not necessary to expose the general pattern of density variation seen in wild-type embryos [12]. Such weighting was only used for the density estimates, not for expression stripe boundary locations. For the average mutant density maps, which were computed using small numbers of embryos, a further reduction in noise was achieved by averaging the left and right halves.

### Modeling nuclear movements from fixed embryo material

The nuclear movement observed in the blastoderm can be decomposed into three components: first, a motion perpendicular to the blastoderm surface that yields a change in the overall 'shape' of the embryo PointCloud where the surface running through the nuclei centers shrinks as the nuclei elongate and move inwards basally; second, a tangential motion, in which the nuclei slide along the surface, pulling closer together or drifting further apart; and third, a global rigid motion, that is, translation and rotation of the entire blastoderm's center of mass and principal axis. The combination of these three yields the three-dimensional motion observed under the microscope. While the relevance of this particular decomposition to the biology is not clear at present, it is convenient for purposes of estimating nuclear motions, which we now describe.

#### Shape change

To compute average shapes for each cohort, the PointCloud surface was parameterized in cylindrical coordinates and the distances from the central axis to nuclei with corresponding (*x*, *φ*) coordinates were averaged together. This yielded an average height map, *H*_*t*_(*x*, *φ*), for each cohort *t *that could then be used to constrain the nuclear placement in estimating nuclear movements. The inward movement of nuclei decreased the apparent total egg length of the PointClouds. This made descriptions of morphological features in terms of percent egg length difficult to accurately compare across different developmental time points since observed changes in features were confounded with a changing coordinate frame. For this reason, embryos were scaled within a cohort to the average length of that cohort rather than unit length.

#### Tangent motion

To model nuclear movements from fixed material, a 'typical early stage embryo' was constructed by placing points in a uniform manner that respected both the average density map and height map computed for stage 5:4-8%. Then, a three-dimensional motion of these points was sought that resulted in the average density and height map measured at stage 5:75-100%. Finding a motion of these 'typical nuclei' is an under-constrained problem (for example, given a motion satisfying the density and height constraints, a new motion in which two nuclei swap locations also satisfies the constraints). Therefore, this problem was regularized by requiring that the motions be small and smooth.

Due to the complicated dependence of density and height on the three-dimensional nuclear locations, a closed-form solution was not readily available. Instead a simple numerical optimization procedure was used to minimize the following cost function:

Let Yit=(xit,φit)
 MathType@MTEF@5@5@+=feaafiart1ev1aaatCvAUfeBSjuyZL2yd9gzLbvyNv2Caerbhv2BYDwAHbqedmvETj2BSbqee0evGueE0jxyaibaiKI8=vI8tuQ8FMI8Gi=hEeeu0xXdbba9frFj0=OqFfea0dXdd9vqai=hGuQ8kuc9pgc9s8qqaq=dirpe0xb9q8qiLsFr0=vr0=vr0dc8meaabaqaciGacaGaaeqabaqadeqadaaakeaatCvAUfKttLearyWrP9MDH5MBPbIqV92AaGabbiab=LfaznaaDaaaleaacaWGPbaabaGaamiDaaaakiabg2da9iaacIcacaWG4bWaa0baaSqaaiaadMgaaeaacaWG0baaaOGaaiilaGGaciab+z8aMnaaDaaaleaacaWGPbaabaGaamiDaaaakiaacMcaaaa@47FF@ be the location of *i*th nucleus in the cylindrical projection coordinates at time *t*. Given an estimate of the average density of nuclei in the cylindrical projection, *D*_*t*_, specified at a set of locations *Z*_*j*_, we seek locations for the nuclei at time *t *which minimizes:

C({Yit})=12∑j‖Dt(Zj)−∑iKσ(‖Zj−Yit‖)‖2+α2∑i≠j‖(Yit−Yit−1)−(Yjt−Yjt−1)‖Yit−1−Yjt−1‖‖2+β2∑i‖Yit−Yit−1‖2
 MathType@MTEF@5@5@+=feaafiart1ev1aaatCvAUfeBSjuyZL2yd9gzLbvyNv2Caerbhv2BYDwAHbqedmvETj2BSbqee0evGueE0jxyaibaiKI8=vI8tuQ8FMI8Gi=hEeeu0xXdbba9frFj0=OqFfea0dXdd9vqai=hGuQ8kuc9pgc9s8qqaq=dirpe0xb9q8qiLsFr0=vr0=vr0dc8meaabaqaciGacaGaaeqabaqadeqadaaakeaacaWGdbGaaiikaiaacUhatCvAUfKttLearyWrP9MDH5MBPbIqV92AaGabbiab=LfaznaaDaaaleaacaWGPbaabaGaamiDaaaakiaac2hacaGGPaGaeyypa0ZaaSaaaeaacaaIXaaabaGaaGOmaaaadaaeqbqaamaafmaabaGaamiramaaBaaaleaacaWG0baabeaakiaacIcacqWFAbGwdaWgaaWcbaGaamOAaaqabaGccaGGPaGaeyOeI0YaaabuaeaacaWGlbWaaSbaaSqaaGGaciab+n8aZbqabaaabaGaamyAaaqab0GaeyyeIuoakiaacIcadaqbdaqaaiab=PfaAnaaBaaaleaacaWGQbaabeaakiabgkHiTiab=LfaznaaDaaaleaacaWGPbaabaGaamiDaaaaaOGaayzcSlaawQa7aiaacMcaaiaawMa7caGLkWoadaahaaWcbeqaaiaaikdaaaaabaGaamOAaaqab0GaeyyeIuoakiabgUcaRmaalaaabaGae4xSdegabaGaaGOmaaaadaaeqbqaamaafmaabaWaaSaaaeaacaGGOaGae8xwaK1aa0baaSqaaiaadMgaaeaacaWG0baaaOGaeyOeI0Iae8xwaK1aa0baaSqaaiaadMgaaeaacaWG0bGaeyOeI0IaaGymaaaakiaacMcacqGHsislcaGGOaGae8xwaK1aa0baaSqaaiaadQgaaeaacaWG0baaaOGaeyOeI0Iae8xwaK1aa0baaSqaaiaadQgaaeaacaWG0bGaeyOeI0IaaGymaaaakiaacMcaaeaadaqbdaqaaiab=LfaznaaDaaaleaacaWGPbaabaGaamiDaiabgkHiTiaaigdaaaGccqGHsislcqWFzbqwdaqhaaWcbaGaamOAaaqaaiaadshacqGHsislcaaIXaaaaaGccaGLjWUaayPcSdaaaaGaayzcSlaawQa7amaaCaaaleqabaGaaGOmaaaaaeaacaWGPbGaeyiyIKRaamOAaaqab0GaeyyeIuoakiabgUcaRmaalaaabaGae4NSdigabaGaaGOmaaaadaaeqbqaamaafmaabaGae8xwaK1aa0baaSqaaiaadMgaaeaacaWG0baaaOGaeyOeI0Iae8xwaK1aa0baaSqaaiaadMgaaeaacaWG0bGaeyOeI0IaaGymaaaaaOGaayzcSlaawQa7amaaCaaaleqabaGaaGOmaaaaaeaacaWGPbaabeqdcqGHris5aaaa@A851@

where the sum of kernels, Kσ(‖Zj−Yit‖)=e−‖Zj−Yit‖2/2σ2
 MathType@MTEF@5@5@+=feaafiart1ev1aaatCvAUfeBSjuyZL2yd9gzLbvyNv2Caerbhv2BYDwAHbqedmvETj2BSbqee0evGueE0jxyaibaiKI8=vI8tuQ8FMI8Gi=hEeeu0xXdbba9frFj0=OqFfea0dXdd9vqai=hGuQ8kuc9pgc9s8qqaq=dirpe0xb9q8qiLsFr0=vr0=vr0dc8meaabaqaciGacaGaaeqabaqadeqadaaakeaacaWGlbWaaSbaaSqaaiabeo8aZbqabaGccaGGOaWaauWaaeaaieqacaWFAbWaaSbaaSqaaiaadQgaaeqaaOGaeyOeI0Iaa8xwamaaDaaaleaacaWGPbaabaGaamiDaaaaaOGaayzcSlaawQa7aiaacMcacqGH9aqpcaWGLbWaaWbaaSqabeaacqGHsisldaqbdaqaaiaa=PfadaWgaaadbaGaamOAaaqabaWccqGHsislcaWFzbWaa0baaWqaaiaadMgaaeaacaWG0baaaaWccaGLjWUaayPcSdWaaWbaaWqabeaacaaIYaaaaSGaai4laiaaikdacqaHdpWCdaahaaadbeqaaiaaikdaaaaaaaaa@5190@, gives a differentiable estimate of the density at point *Z*_*j *_as a function of the placement of the nuclei {Yit
 MathType@MTEF@5@5@+=feaafiart1ev1aaatCvAUfeBSjuyZL2yd9gzLbvyNv2Caerbhv2BYDwAHbqedmvETj2BSbqee0evGueE0jxyaibaiKI8=vI8tuQ8FMI8Gi=hEeeu0xXdbba9frFj0=OqFfea0dXdd9vqai=hGuQ8kuc9pgc9s8qqaq=dirpe0xb9q8qiLsFr0=vr0=vr0dc8meaabaqaciGacaGaaeqabaqadeqadaaakeaaieqacaWFzbWaa0baaSqaaiaadMgaaeaacaWG0baaaaaa@3625@} [44].

The first term in the cost function is a data fidelity term that penalizes mismatch between the average measured density and the density resulting from the current placement of the synthetic nuclei. The second term penalizes the extent to which nearby cells' motion vectors are allowed to differ. The third term penalizes large nuclear movements. *α *and *β *specify the relative importance of the two regularization terms. Once appropriate minimizing cylindrical coordinates had been found for each nucleus, the average cohort height map immediately yielded three-dimensional locations as a function of the cylindrical coordinates that automatically satisfied the shape constraints.

The cost function was optimized using a standard conjugate gradient routine [45]. In the case of the early stage nuclear placement, the optimization was initialized with a uniform grid of points and only the first cost term was used. For the late stage placement, the solution from the early stage placement was used as initialization. In both cases, conjugate gradient converged to reasonable solutions within a few hundred line-searches. The resulting motion predictions were quite insensitive to the relative weighting of the three cost terms and robust to perturbations of the initial conditions. The only degeneracy observed was for small smoothness penalties and extreme initial conditions consisting of large swirls or vortices that resulted in convergence to qualitatively different local minima.

One shortfall of the optimization routine described above is that the cylindrical parameterization has singularities at the poles of the embryo. These singularities can be removed by working in multiple coordinate charts simultaneously and using a cost function that smoothly blends between costs defined in each coordinate chart. In the final results presented here, we used such a scheme with one spherical coordinate system covering the middle 80% of the embryo and two spherical charts to capture the remaining 10% at each pole. For additional details see [46,47].

#### Global rigid motion

Unfortunately, it was impossible to extract any global rigid transformation from the fixed material since there was no absolute coordinate frame from which to judge translation or rotation (that is, embryos are placed in an arbitrary location on the slide). To set the translation between the early and late stage synthetic embryos, the smaller, late-stage synthetic embryo was located so as to minimize the Hausdorff distance between the two surfaces, so that no point on the late synthetic embryo was far from its closest point on the early synthetic embryo (aligning the center of mass of the two point clouds yielded a similar translation). It was assumed that there is no global rotation. The resulting placement matches the observation made in our live imaging experiments in that the inward nuclear movement was fairly uniform over the entire blastoderm surface relative to the vitelline membrane.

### Modeling the contributions of expression flow and nuclear flow

The canonical early and late synthetic embryos were then used to untangle the relative contributions of expression flow and nuclear flow to pattern flow. The locations of stripe boundaries in three dimensions for the early stage cohort were extracted as in [12]. The three-dimensional motion field given by the synthetic nuclei was interpolated to yield a predicted motion undergone by the stripe boundary in the absence of expression flow. The final resting place of each stripe boundary was then compared to the observed late stage stripe location.

It should be noted that, when the flow fields from the live embryos (Figure 2c) were applied to the early synthetic embryo, the nuclear densities developed into patterns different from those observed in late fixed embryos. This could have resulted, for example, from strain-specific differences in movement between the fixed CantonS wild-type embryos and Histone2-GFP embryos, or from inaccuracies inherent in the live embryo data. Thus, although our model of cell movement recapitulates the same patterns of nuclear density and blastoderm shape measured in wild-type embryos, the flow maps need some further verification.

## Additional data files

The following additional data are available with the online version of this paper. Additional data file 1 contains all TIFF files used to analyze nuclear movements in 22 living Histone2A-GFP embryos.

## Supplementary Material

Additional data file 1 All TIFF files used to analyze nuclear movements in 22 living Histone2A-GFP embryos. Click here for file

## Figures and Tables

**Table 1:** The mean number of nuclei in wild-type PointClouds

	Stage cohort					
	
	5:0-3%	5:4-8%	5:9-25%	5:26-50%	5:51-75%	5:76-100%
No. of embryos	144	153	146	151	170	130
Mean no. of nuclei	6,065	6,079	6,095	6,093	5,997	5,898
SD no. of nuclei	334	249	266	241	281	285
95% CI	± 55	± 39	± 43	± 38	± 42	± 49

The standard deviation (SD) and 95% confidence intervals (CI) for the mean are shown for each of the temporal cohorts studied. The last two cohorts have lower numbers of nuclei, probably due to segmentation errors affecting data from increasingly dense dorsal regions (see [12]). Because the local nuclear density differences develop well before the embryos have reached these last two temporal cohorts, we conclude that the blastoderm density changes are due to nuclear movement, not the preferential loss or increase of nuclei.
