# Supplementary figures and images for: Three-dimensional morphology and gene expression in the Drosophila blastoderm at cellular resolution II: dynamics (part 2 of 2)
Source: Genome Biol. 2006 Dec 21;7(12):R124. doi: 10.1186/gb-2006-7-12-r124 (PMC1794437; doi:10.1186/gb-2006-7-12-r124)

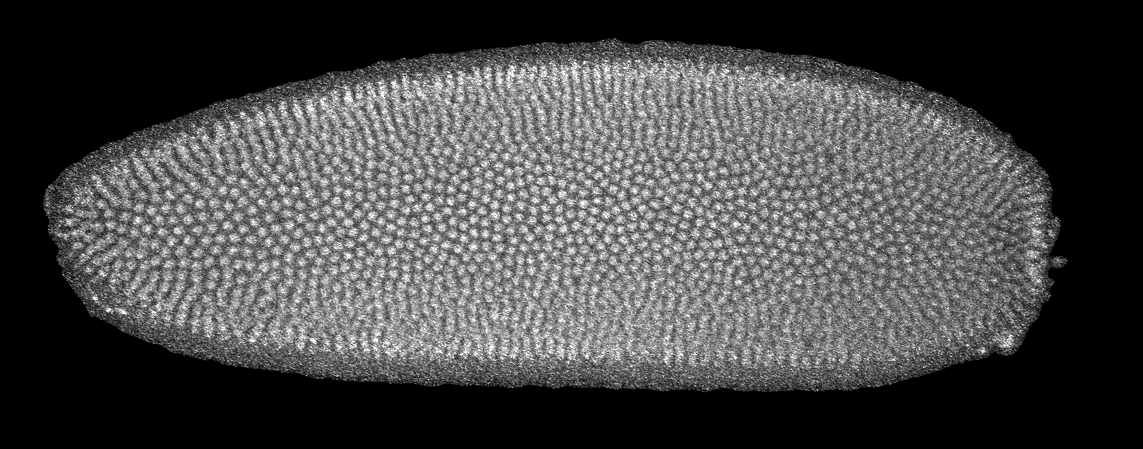

Supplement: Additional data file 1 — All TIFF files used to analyze nuclear movements in 22 living Histone2A-GFP embryos. [file gb-2006-7-12-r124-S1.zip › 150705e4/step-01.tif]

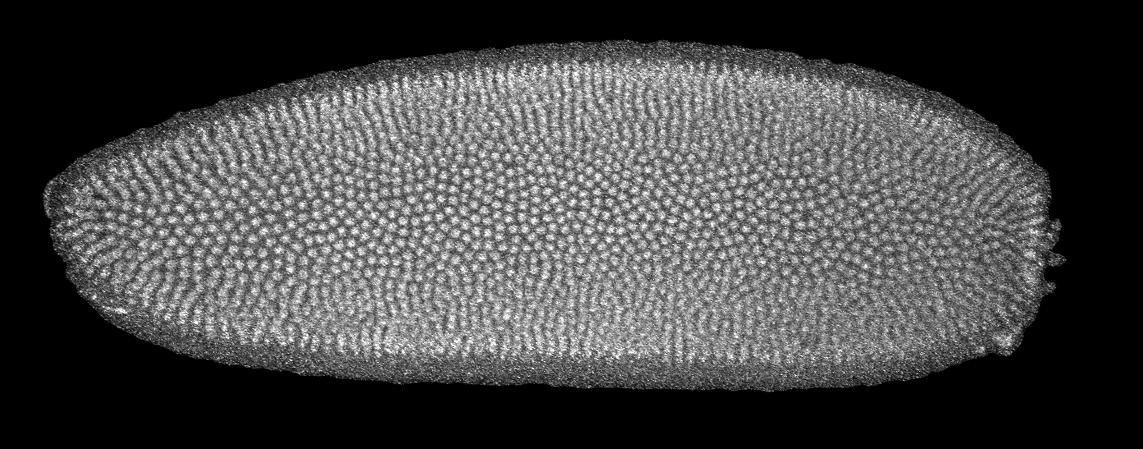

Supplement: Additional data file 1 — All TIFF files used to analyze nuclear movements in 22 living Histone2A-GFP embryos. [file gb-2006-7-12-r124-S1.zip › 150705e4/step-02.tif]

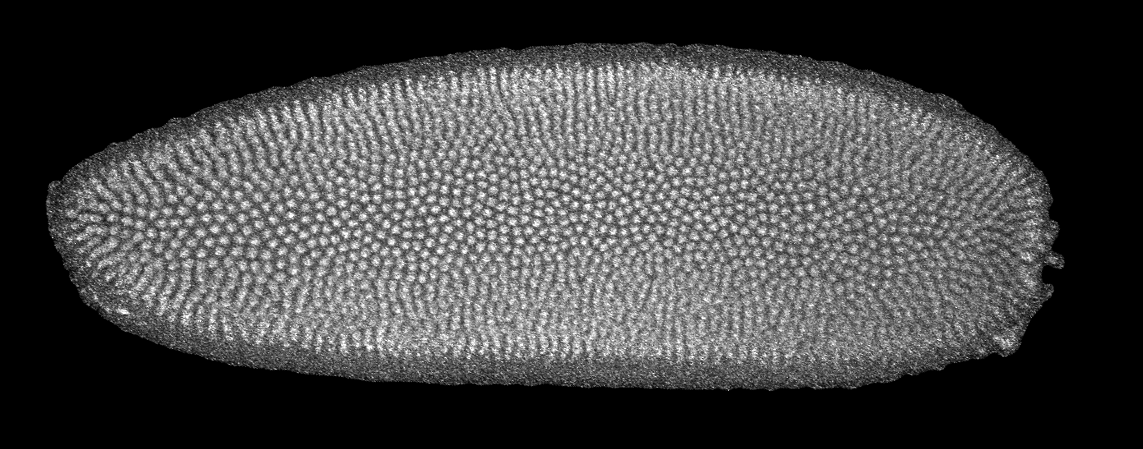

Supplement: Additional data file 1 — All TIFF files used to analyze nuclear movements in 22 living Histone2A-GFP embryos. [file gb-2006-7-12-r124-S1.zip › 150705e4/step-03.tif]

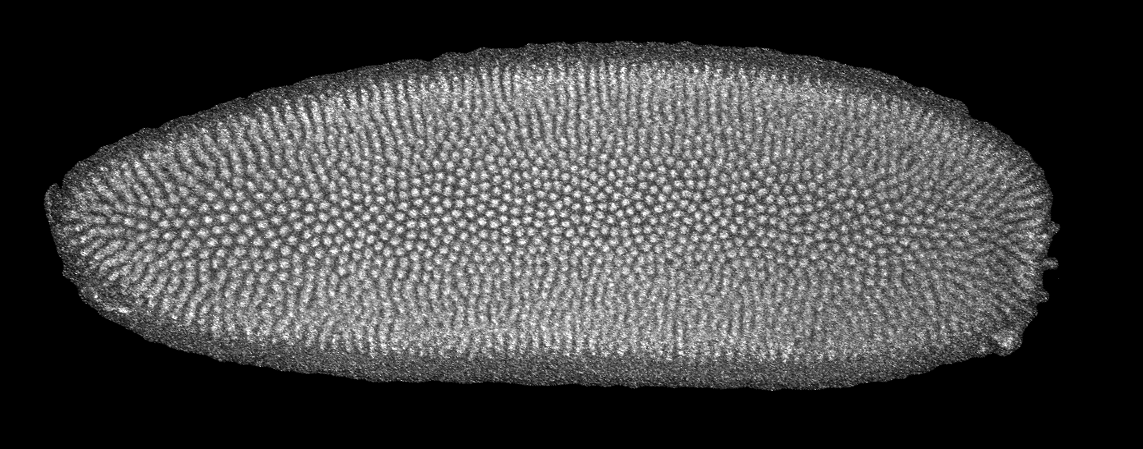

Supplement: Additional data file 1 — All TIFF files used to analyze nuclear movements in 22 living Histone2A-GFP embryos. [file gb-2006-7-12-r124-S1.zip › 150705e4/step-04.tif]

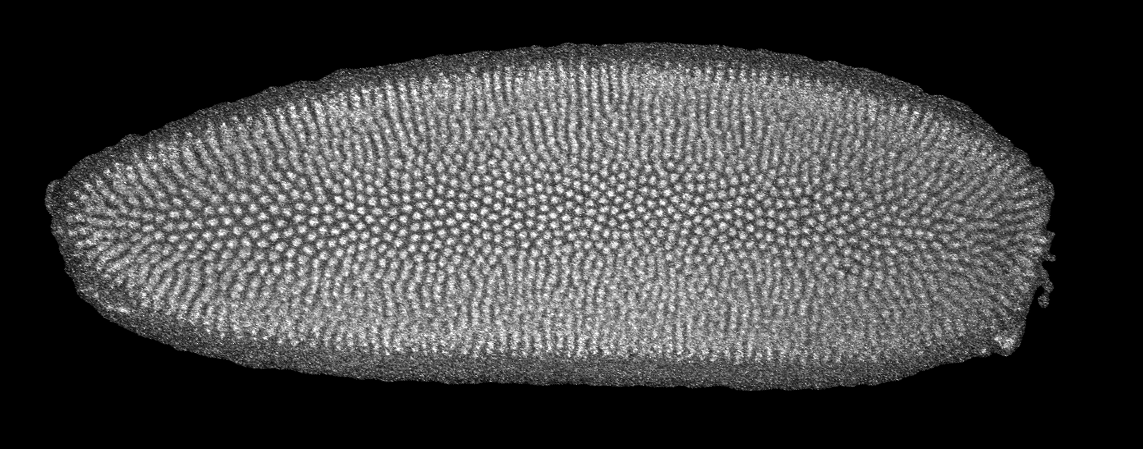

Supplement: Additional data file 1 — All TIFF files used to analyze nuclear movements in 22 living Histone2A-GFP embryos. [file gb-2006-7-12-r124-S1.zip › 150705e4/step-05.tif]

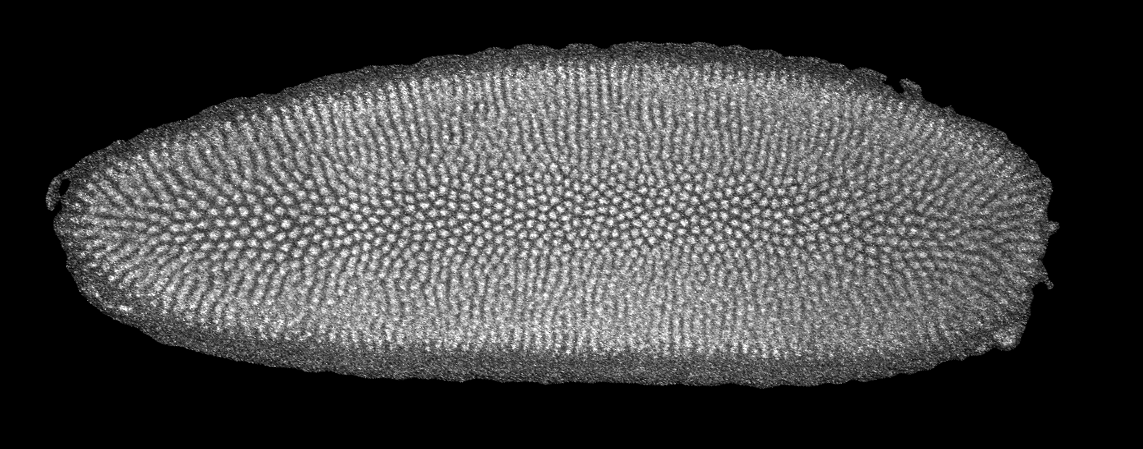

Supplement: Additional data file 1 — All TIFF files used to analyze nuclear movements in 22 living Histone2A-GFP embryos. [file gb-2006-7-12-r124-S1.zip › 150705e4/step-06.tif]

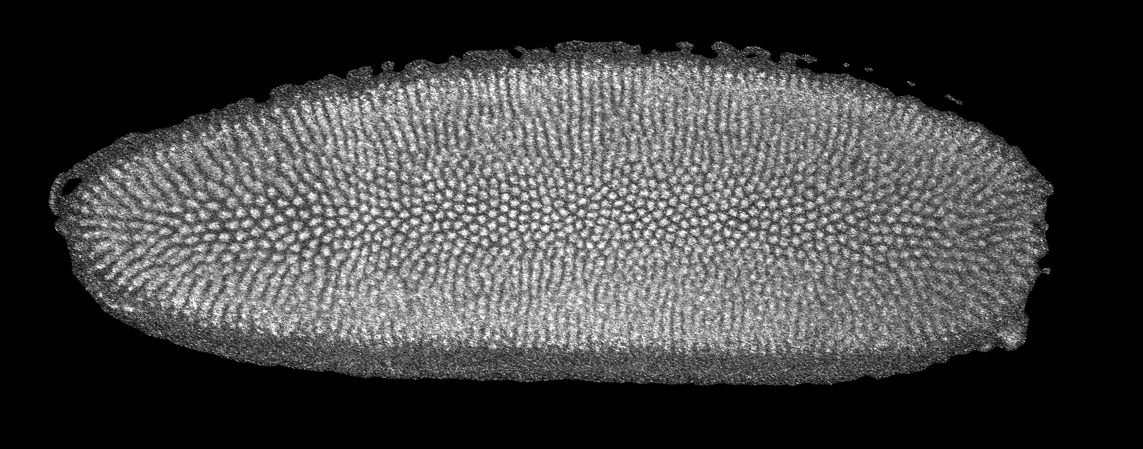

Supplement: Additional data file 1 — All TIFF files used to analyze nuclear movements in 22 living Histone2A-GFP embryos. [file gb-2006-7-12-r124-S1.zip › 150705e4/step-07.tif]

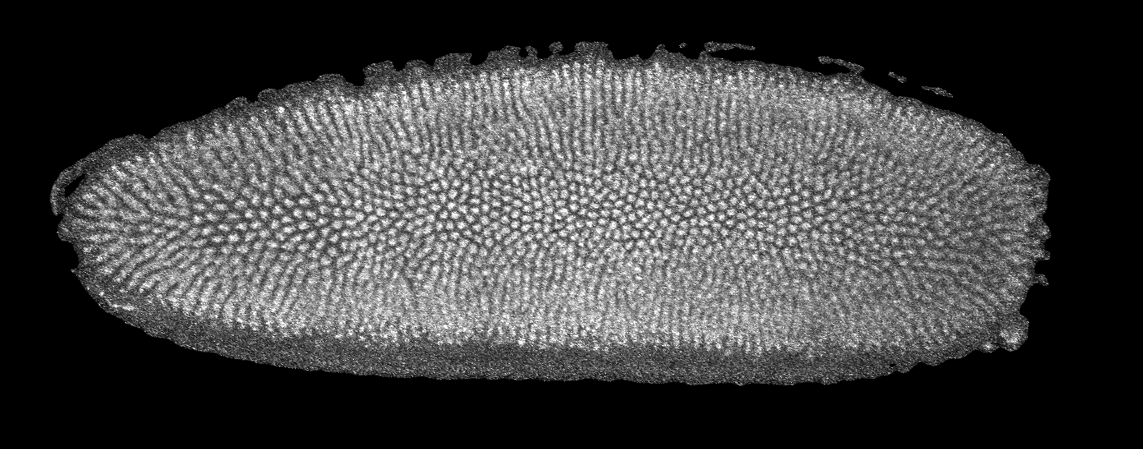

Supplement: Additional data file 1 — All TIFF files used to analyze nuclear movements in 22 living Histone2A-GFP embryos. [file gb-2006-7-12-r124-S1.zip › 150705e4/step-08.tif]

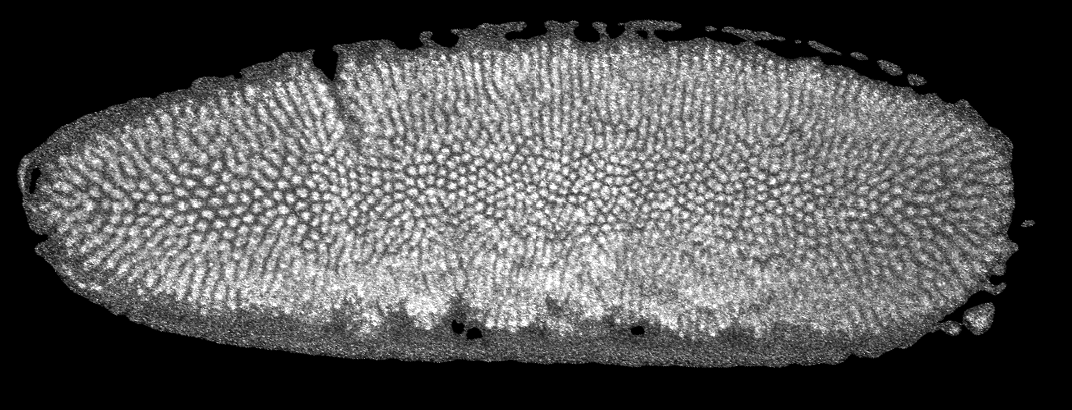

Supplement: Additional data file 1 — All TIFF files used to analyze nuclear movements in 22 living Histone2A-GFP embryos. [file gb-2006-7-12-r124-S1.zip › 150705e4/gastrulated.tif]

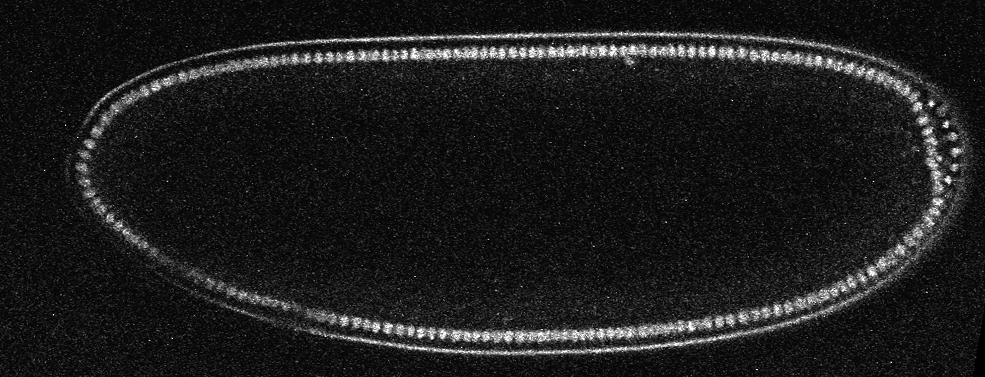

Supplement: Additional data file 1 — All TIFF files used to analyze nuclear movements in 22 living Histone2A-GFP embryos. [file gb-2006-7-12-r124-S1.zip › 151005e3/slice-0.tif]

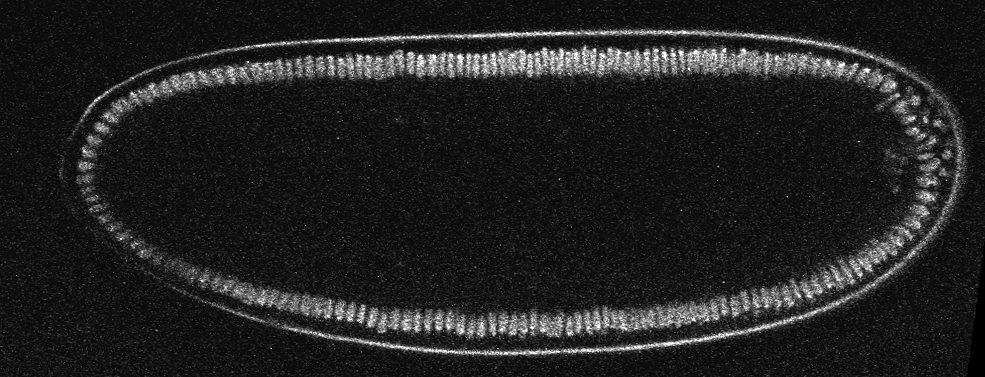

Supplement: Additional data file 1 — All TIFF files used to analyze nuclear movements in 22 living Histone2A-GFP embryos. [file gb-2006-7-12-r124-S1.zip › 151005e3/slice-1.tif]

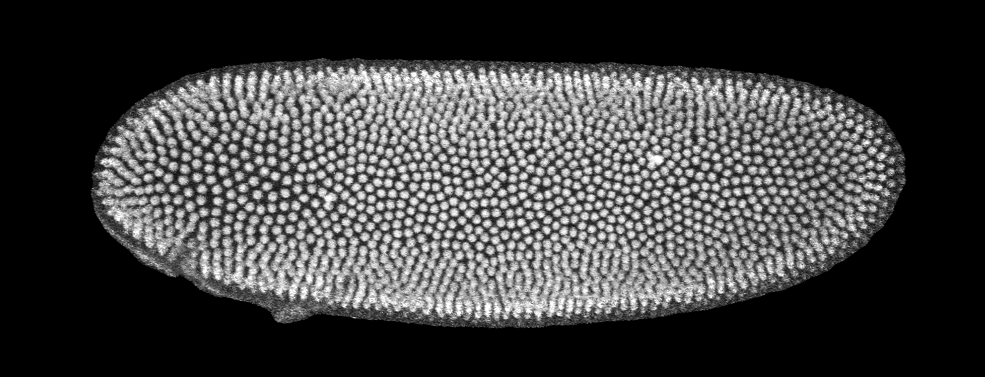

Supplement: Additional data file 1 — All TIFF files used to analyze nuclear movements in 22 living Histone2A-GFP embryos. [file gb-2006-7-12-r124-S1.zip › 151005e3/step-00.tif]

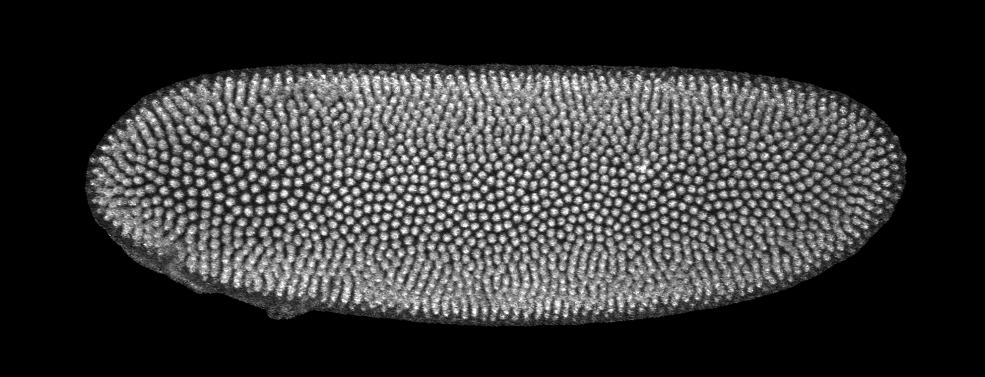

Supplement: Additional data file 1 — All TIFF files used to analyze nuclear movements in 22 living Histone2A-GFP embryos. [file gb-2006-7-12-r124-S1.zip › 151005e3/step-01.tif]

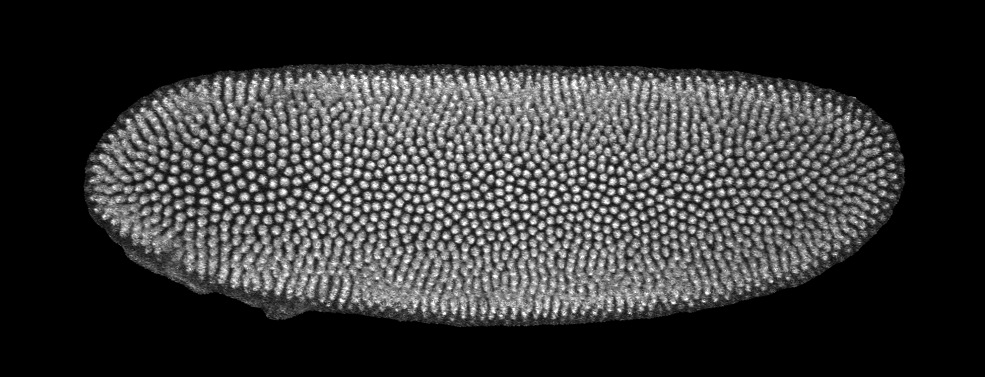

Supplement: Additional data file 1 — All TIFF files used to analyze nuclear movements in 22 living Histone2A-GFP embryos. [file gb-2006-7-12-r124-S1.zip › 151005e3/step-02.tif]

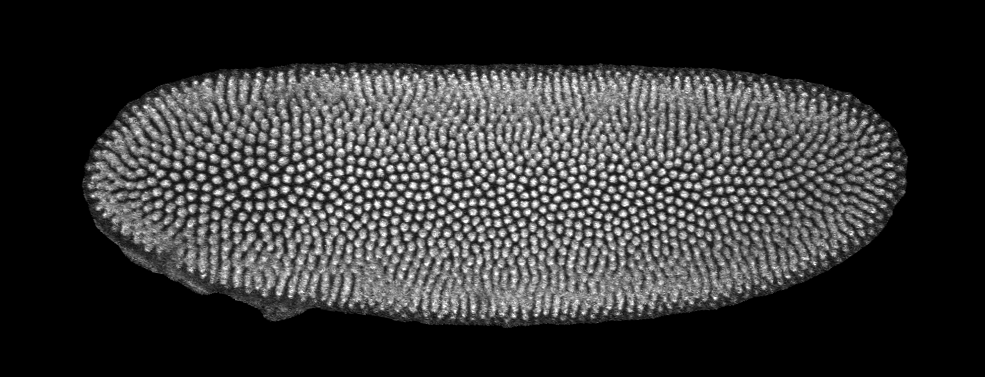

Supplement: Additional data file 1 — All TIFF files used to analyze nuclear movements in 22 living Histone2A-GFP embryos. [file gb-2006-7-12-r124-S1.zip › 151005e3/step-03.tif]

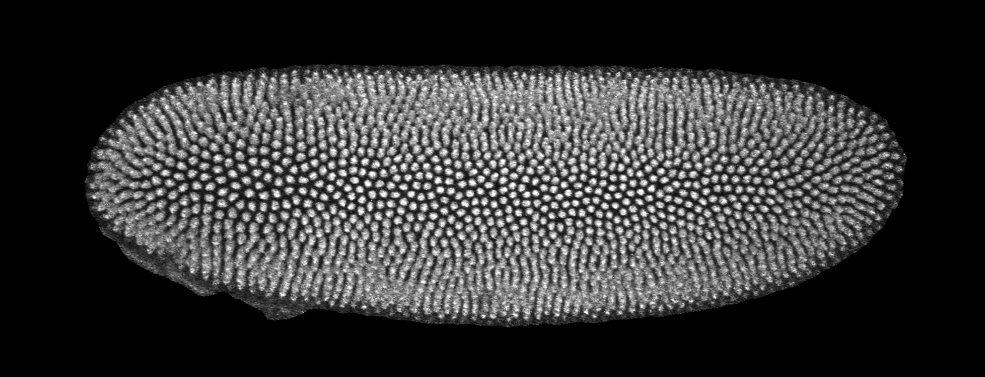

Supplement: Additional data file 1 — All TIFF files used to analyze nuclear movements in 22 living Histone2A-GFP embryos. [file gb-2006-7-12-r124-S1.zip › 151005e3/step-04.tif]

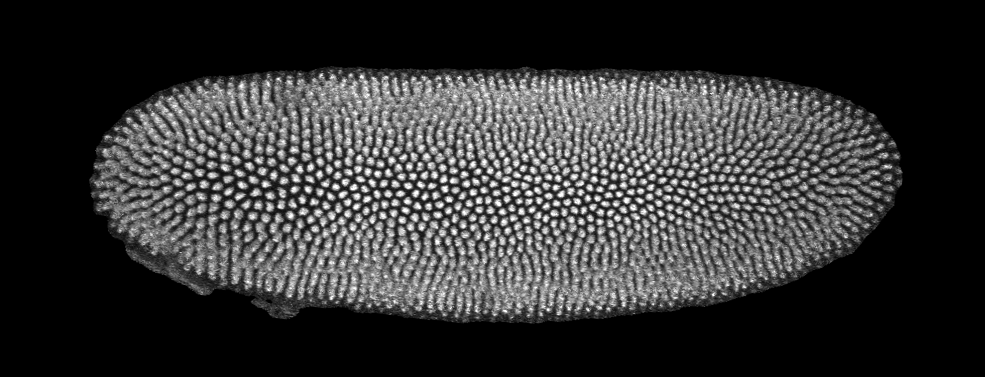

Supplement: Additional data file 1 — All TIFF files used to analyze nuclear movements in 22 living Histone2A-GFP embryos. [file gb-2006-7-12-r124-S1.zip › 151005e3/step-05.tif]

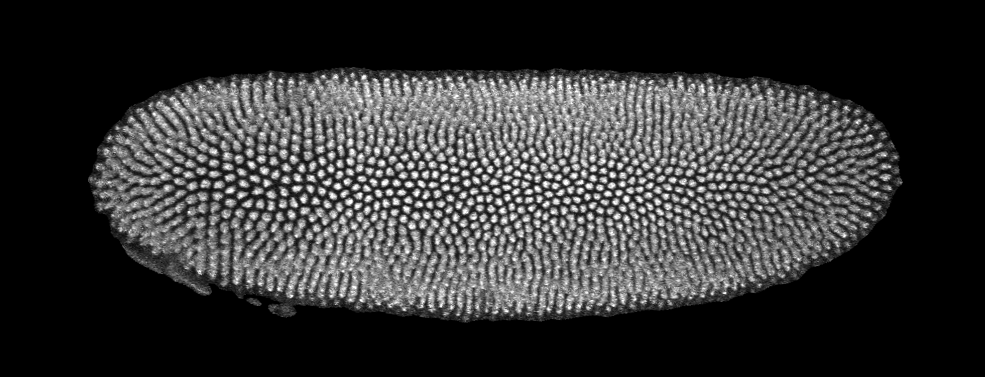

Supplement: Additional data file 1 — All TIFF files used to analyze nuclear movements in 22 living Histone2A-GFP embryos. [file gb-2006-7-12-r124-S1.zip › 151005e3/step-06.tif]

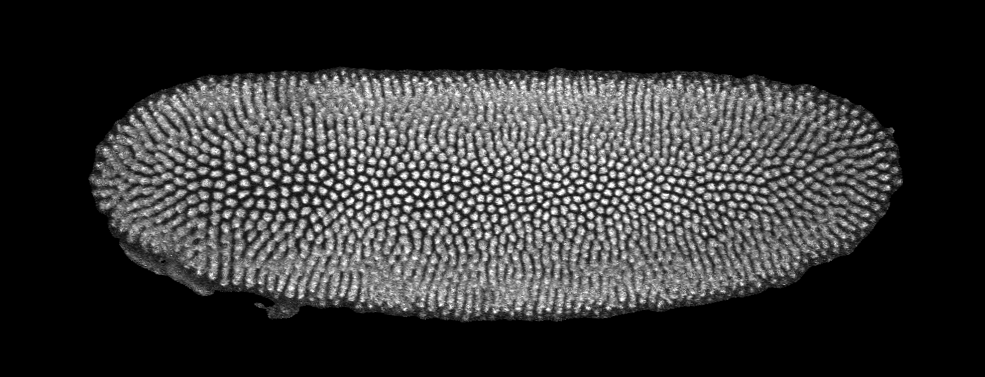

Supplement: Additional data file 1 — All TIFF files used to analyze nuclear movements in 22 living Histone2A-GFP embryos. [file gb-2006-7-12-r124-S1.zip › 151005e3/step-07.tif]

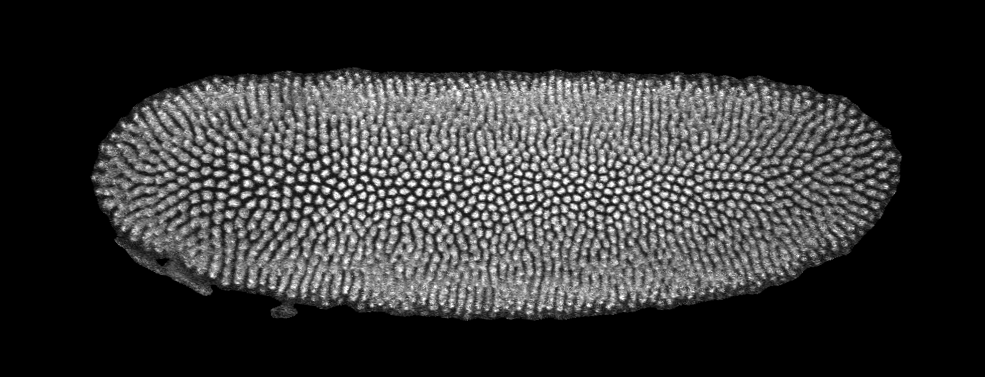

Supplement: Additional data file 1 — All TIFF files used to analyze nuclear movements in 22 living Histone2A-GFP embryos. [file gb-2006-7-12-r124-S1.zip › 151005e3/step-08.tif]

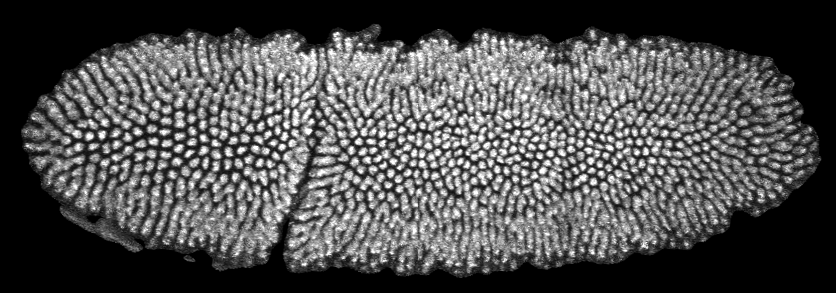

Supplement: Additional data file 1 — All TIFF files used to analyze nuclear movements in 22 living Histone2A-GFP embryos. [file gb-2006-7-12-r124-S1.zip › 151005e3/gastrulated.tif]

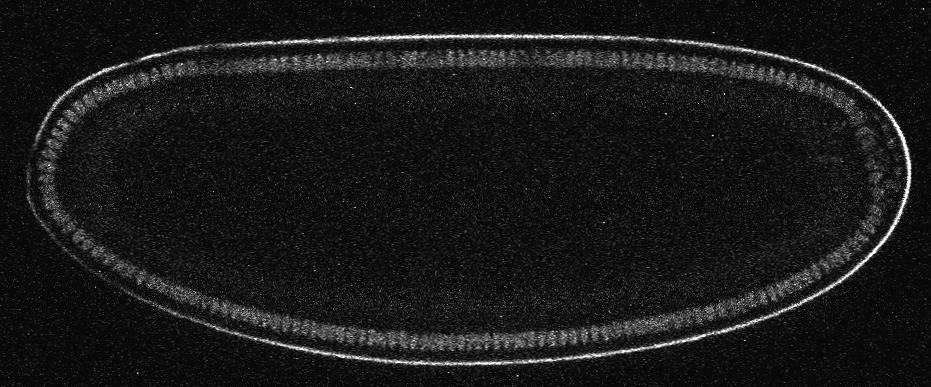

Supplement: Additional data file 1 — All TIFF files used to analyze nuclear movements in 22 living Histone2A-GFP embryos. [file gb-2006-7-12-r124-S1.zip › 151005e4/slice-0.tif]

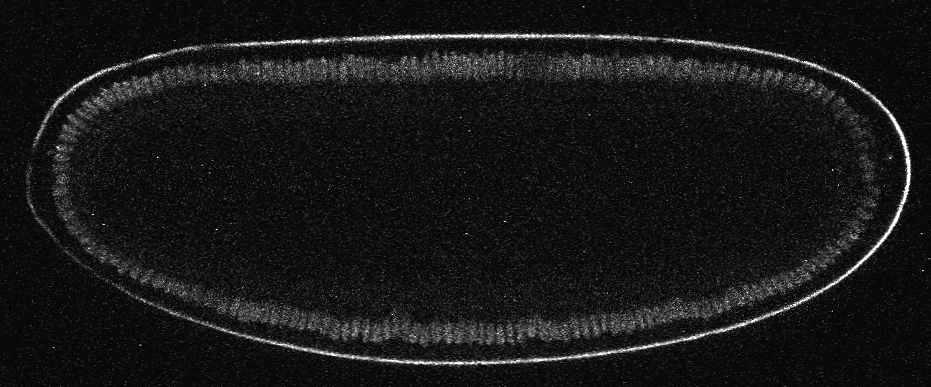

Supplement: Additional data file 1 — All TIFF files used to analyze nuclear movements in 22 living Histone2A-GFP embryos. [file gb-2006-7-12-r124-S1.zip › 151005e4/slice-1.tif]

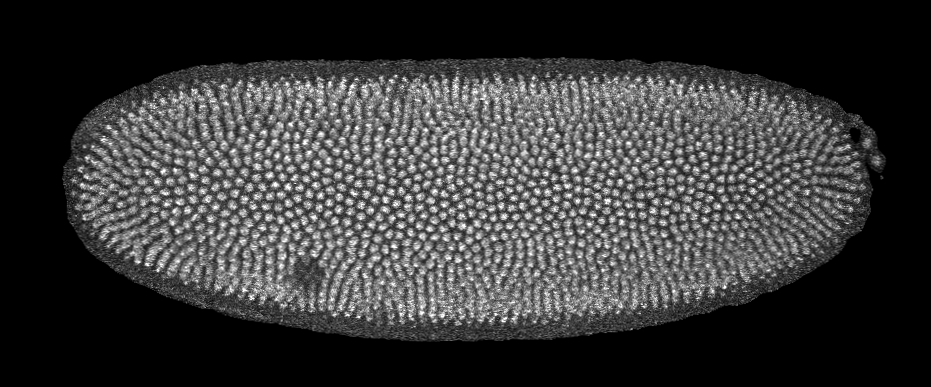

Supplement: Additional data file 1 — All TIFF files used to analyze nuclear movements in 22 living Histone2A-GFP embryos. [file gb-2006-7-12-r124-S1.zip › 151005e4/step-00.tif]

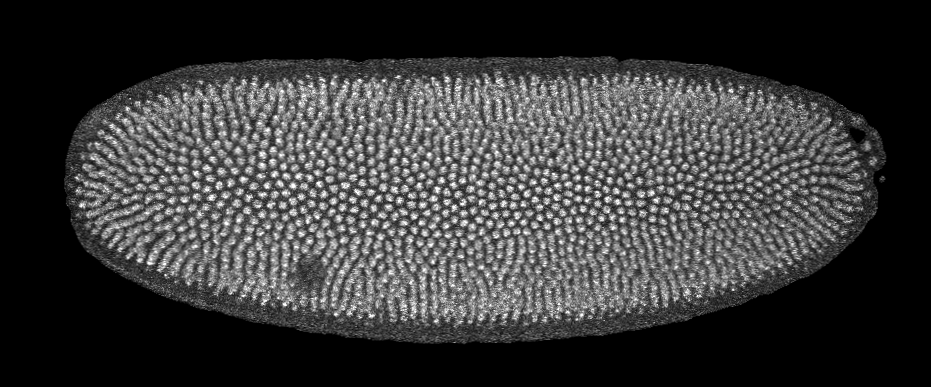

Supplement: Additional data file 1 — All TIFF files used to analyze nuclear movements in 22 living Histone2A-GFP embryos. [file gb-2006-7-12-r124-S1.zip › 151005e4/step-01.tif]

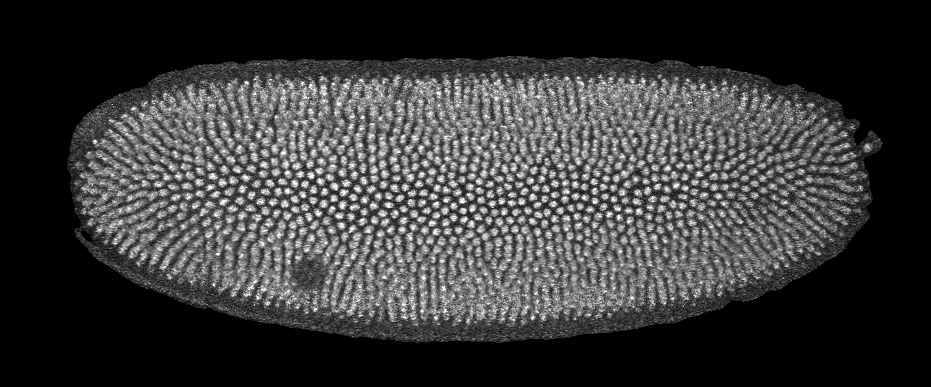

Supplement: Additional data file 1 — All TIFF files used to analyze nuclear movements in 22 living Histone2A-GFP embryos. [file gb-2006-7-12-r124-S1.zip › 151005e4/step-02.tif]

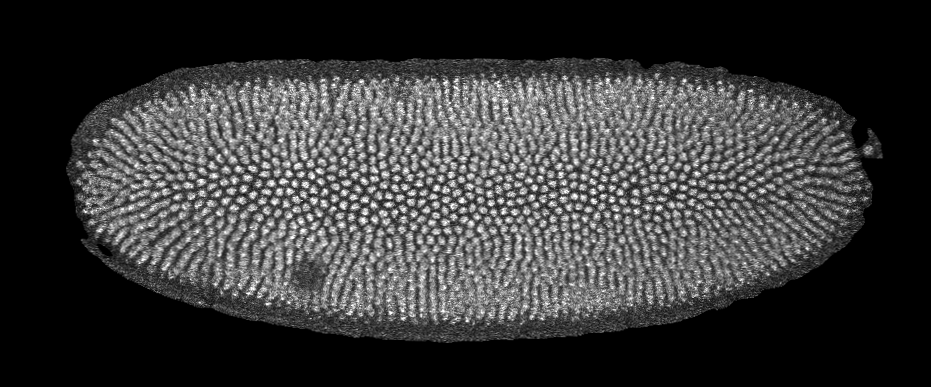

Supplement: Additional data file 1 — All TIFF files used to analyze nuclear movements in 22 living Histone2A-GFP embryos. [file gb-2006-7-12-r124-S1.zip › 151005e4/step-03.tif]

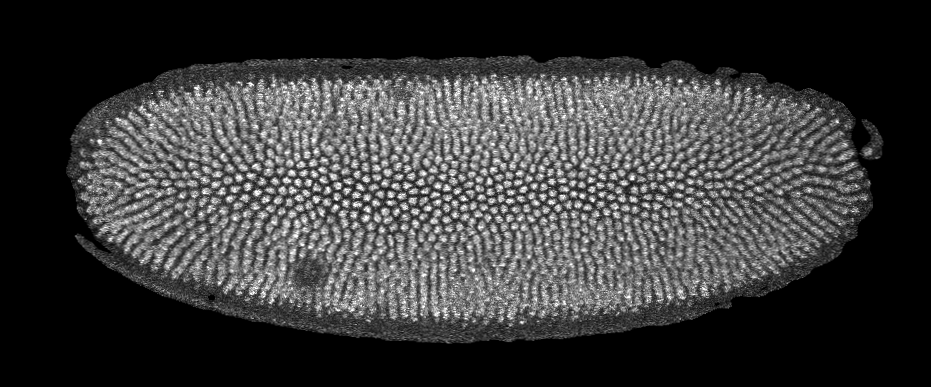

Supplement: Additional data file 1 — All TIFF files used to analyze nuclear movements in 22 living Histone2A-GFP embryos. [file gb-2006-7-12-r124-S1.zip › 151005e4/step-04.tif]

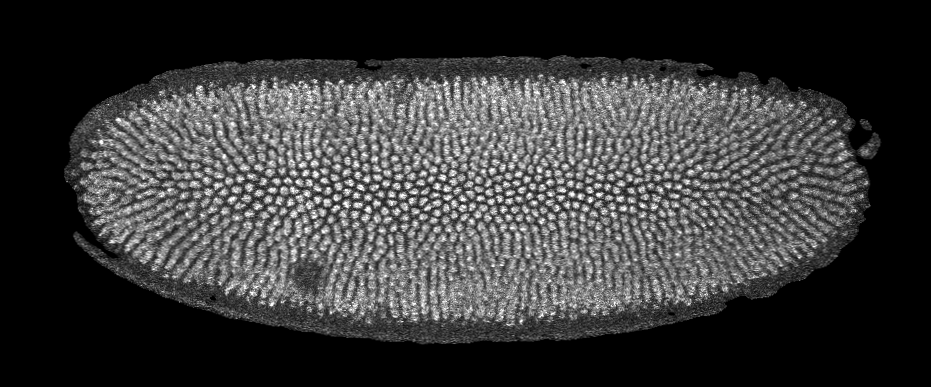

Supplement: Additional data file 1 — All TIFF files used to analyze nuclear movements in 22 living Histone2A-GFP embryos. [file gb-2006-7-12-r124-S1.zip › 151005e4/step-05.tif]

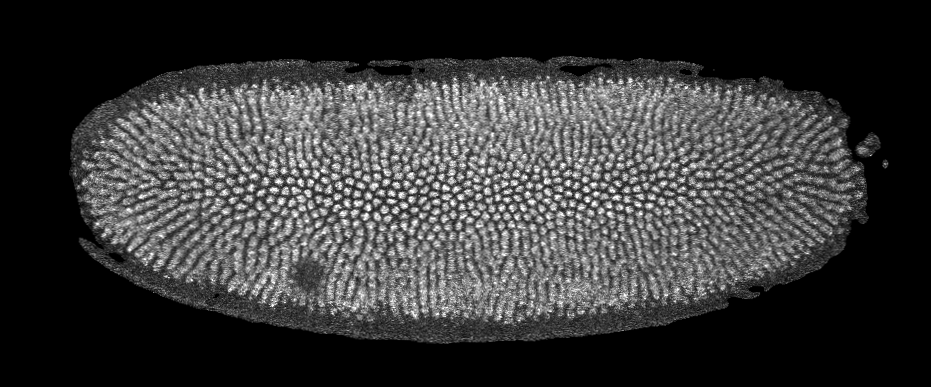

Supplement: Additional data file 1 — All TIFF files used to analyze nuclear movements in 22 living Histone2A-GFP embryos. [file gb-2006-7-12-r124-S1.zip › 151005e4/step-06.tif]

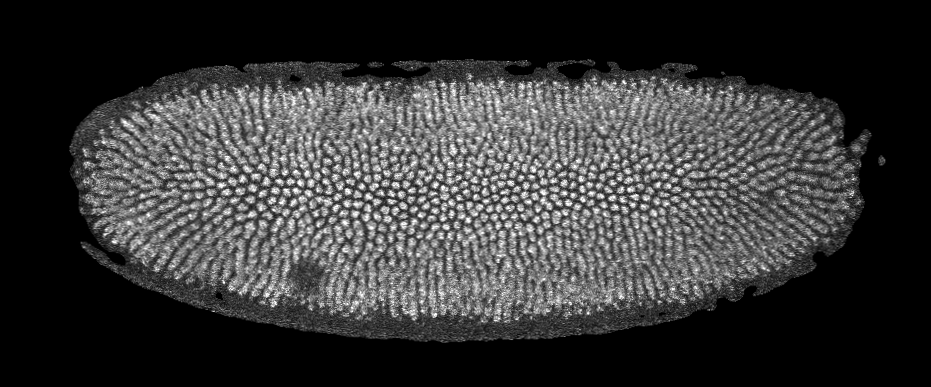

Supplement: Additional data file 1 — All TIFF files used to analyze nuclear movements in 22 living Histone2A-GFP embryos. [file gb-2006-7-12-r124-S1.zip › 151005e4/step-07.tif]

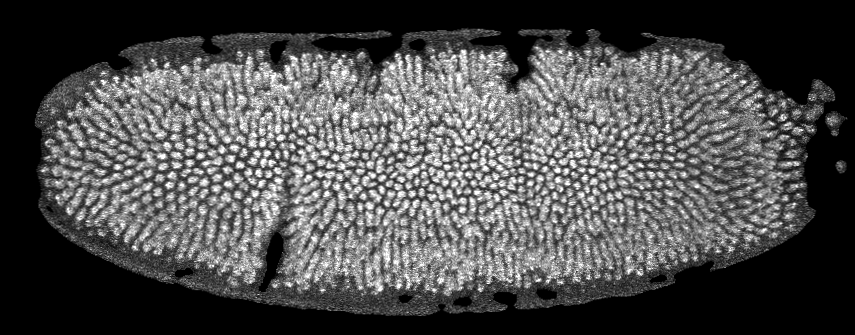

Supplement: Additional data file 1 — All TIFF files used to analyze nuclear movements in 22 living Histone2A-GFP embryos. [file gb-2006-7-12-r124-S1.zip › 151005e4/gastrulated.tif]

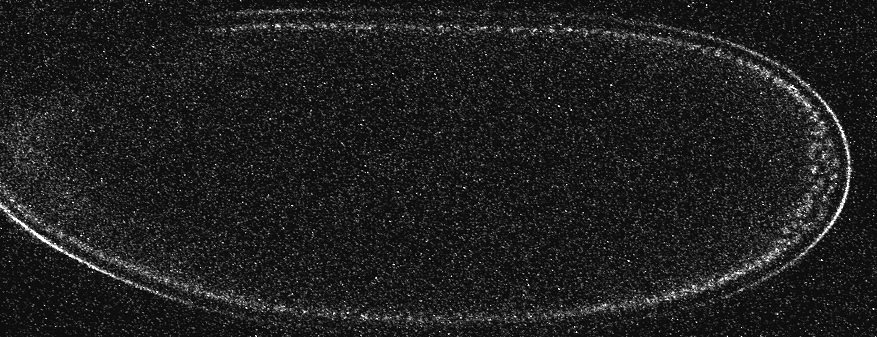

Supplement: Additional data file 1 — All TIFF files used to analyze nuclear movements in 22 living Histone2A-GFP embryos. [file gb-2006-7-12-r124-S1.zip › 171105e2/slice-0.tif]

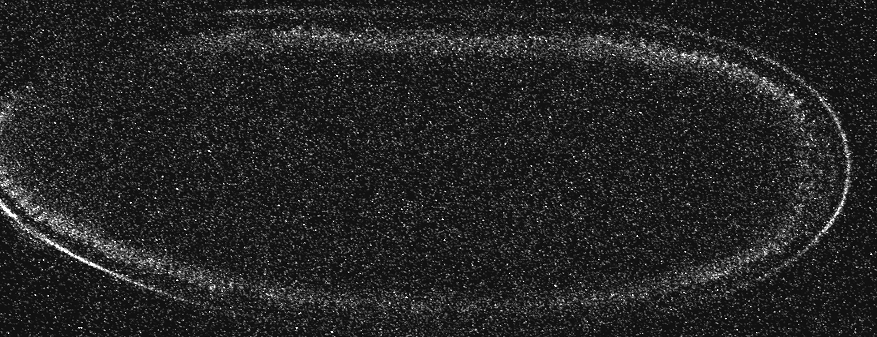

Supplement: Additional data file 1 — All TIFF files used to analyze nuclear movements in 22 living Histone2A-GFP embryos. [file gb-2006-7-12-r124-S1.zip › 171105e2/slice-1.tif]

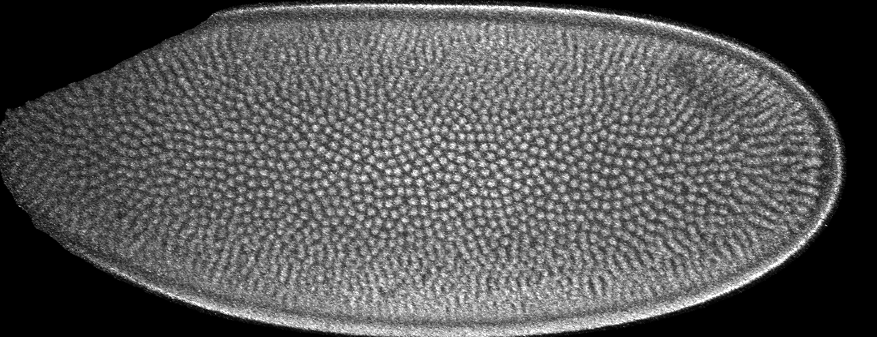

Supplement: Additional data file 1 — All TIFF files used to analyze nuclear movements in 22 living Histone2A-GFP embryos. [file gb-2006-7-12-r124-S1.zip › 171105e2/step-00.tif]

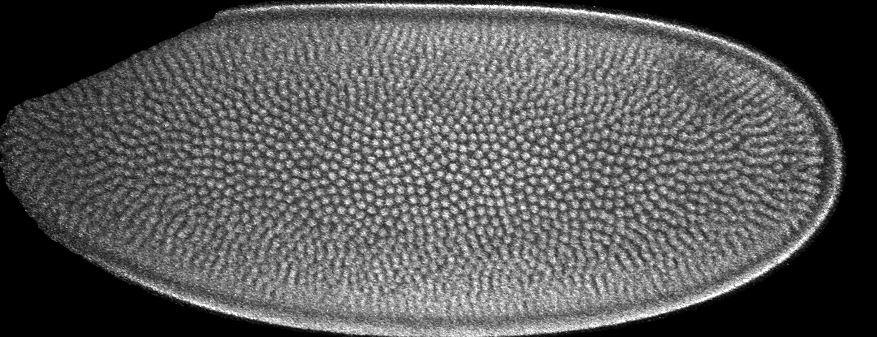

Supplement: Additional data file 1 — All TIFF files used to analyze nuclear movements in 22 living Histone2A-GFP embryos. [file gb-2006-7-12-r124-S1.zip › 171105e2/step-01.tif]

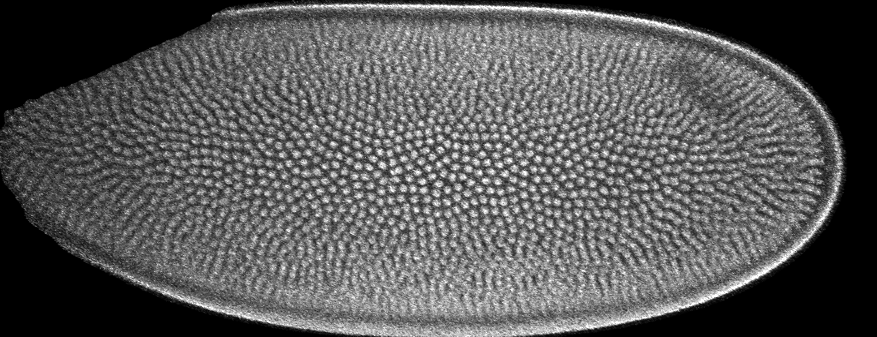

Supplement: Additional data file 1 — All TIFF files used to analyze nuclear movements in 22 living Histone2A-GFP embryos. [file gb-2006-7-12-r124-S1.zip › 171105e2/step-02.tif]

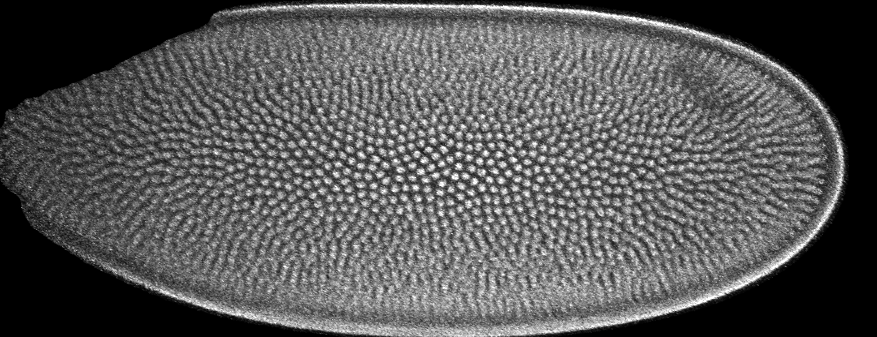

Supplement: Additional data file 1 — All TIFF files used to analyze nuclear movements in 22 living Histone2A-GFP embryos. [file gb-2006-7-12-r124-S1.zip › 171105e2/step-03.tif]

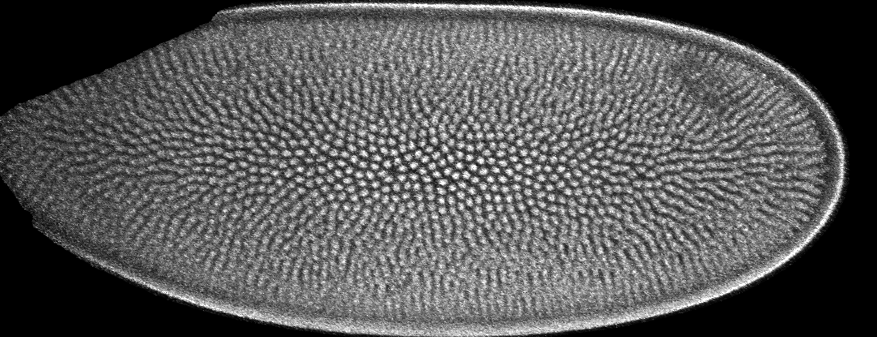

Supplement: Additional data file 1 — All TIFF files used to analyze nuclear movements in 22 living Histone2A-GFP embryos. [file gb-2006-7-12-r124-S1.zip › 171105e2/step-04.tif]

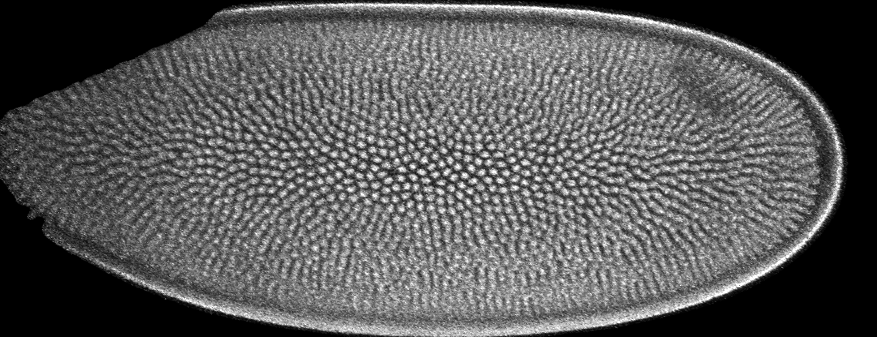

Supplement: Additional data file 1 — All TIFF files used to analyze nuclear movements in 22 living Histone2A-GFP embryos. [file gb-2006-7-12-r124-S1.zip › 171105e2/step-05.tif]

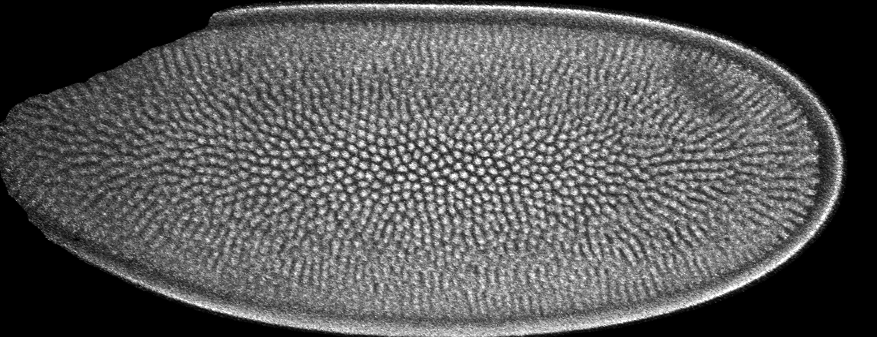

Supplement: Additional data file 1 — All TIFF files used to analyze nuclear movements in 22 living Histone2A-GFP embryos. [file gb-2006-7-12-r124-S1.zip › 171105e2/step-06.tif]

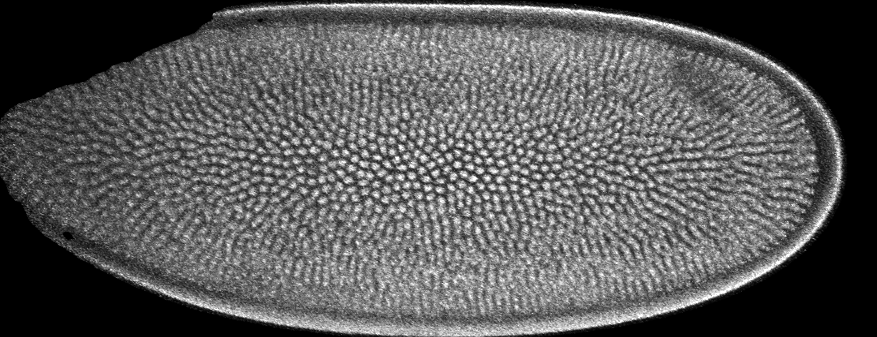

Supplement: Additional data file 1 — All TIFF files used to analyze nuclear movements in 22 living Histone2A-GFP embryos. [file gb-2006-7-12-r124-S1.zip › 171105e2/step-07.tif]

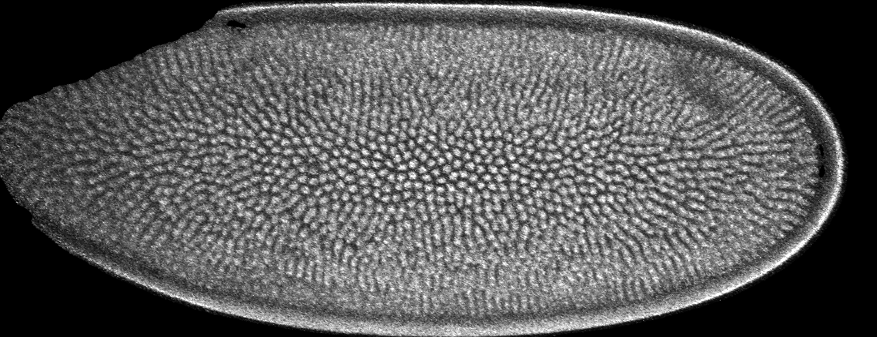

Supplement: Additional data file 1 — All TIFF files used to analyze nuclear movements in 22 living Histone2A-GFP embryos. [file gb-2006-7-12-r124-S1.zip › 171105e2/step-08.tif]

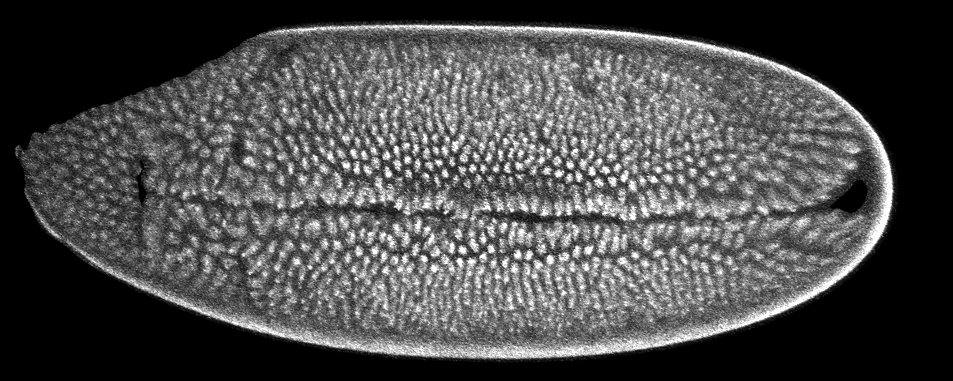

Supplement: Additional data file 1 — All TIFF files used to analyze nuclear movements in 22 living Histone2A-GFP embryos. [file gb-2006-7-12-r124-S1.zip › 171105e2/gastrulated.tif]

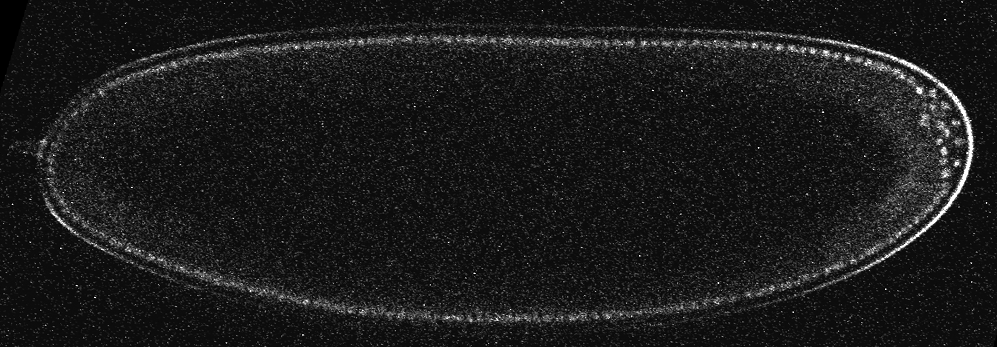

Supplement: Additional data file 1 — All TIFF files used to analyze nuclear movements in 22 living Histone2A-GFP embryos. [file gb-2006-7-12-r124-S1.zip › 171105e3/slice-0.tif]

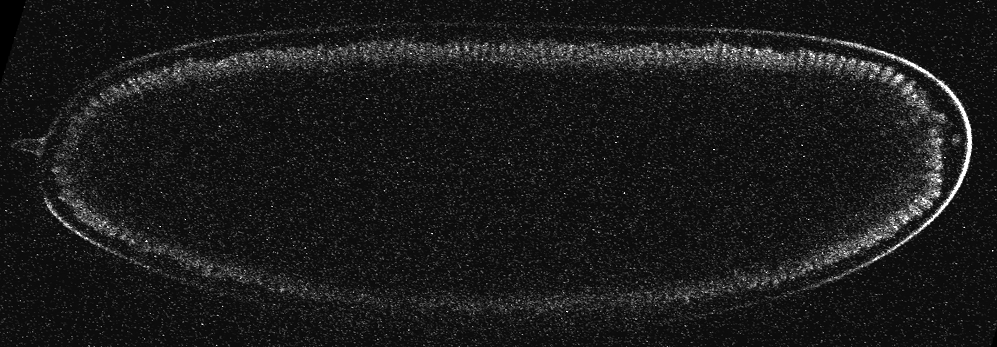

Supplement: Additional data file 1 — All TIFF files used to analyze nuclear movements in 22 living Histone2A-GFP embryos. [file gb-2006-7-12-r124-S1.zip › 171105e3/slice-1.tif]

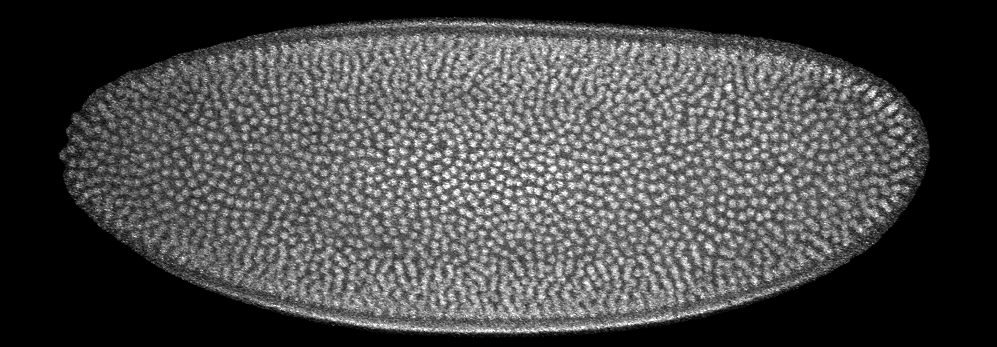

Supplement: Additional data file 1 — All TIFF files used to analyze nuclear movements in 22 living Histone2A-GFP embryos. [file gb-2006-7-12-r124-S1.zip › 171105e3/step-00.tif]

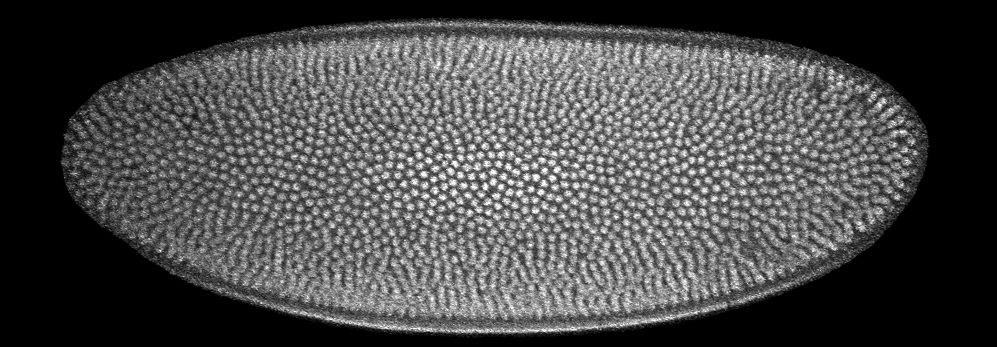

Supplement: Additional data file 1 — All TIFF files used to analyze nuclear movements in 22 living Histone2A-GFP embryos. [file gb-2006-7-12-r124-S1.zip › 171105e3/step-01.tif]

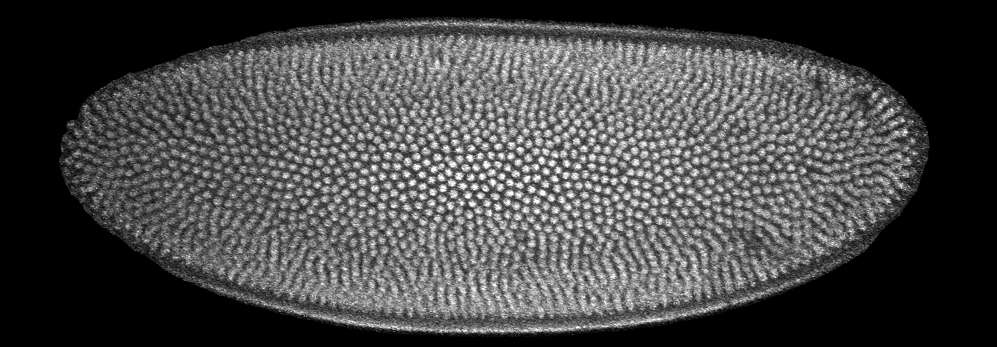

Supplement: Additional data file 1 — All TIFF files used to analyze nuclear movements in 22 living Histone2A-GFP embryos. [file gb-2006-7-12-r124-S1.zip › 171105e3/step-02.tif]

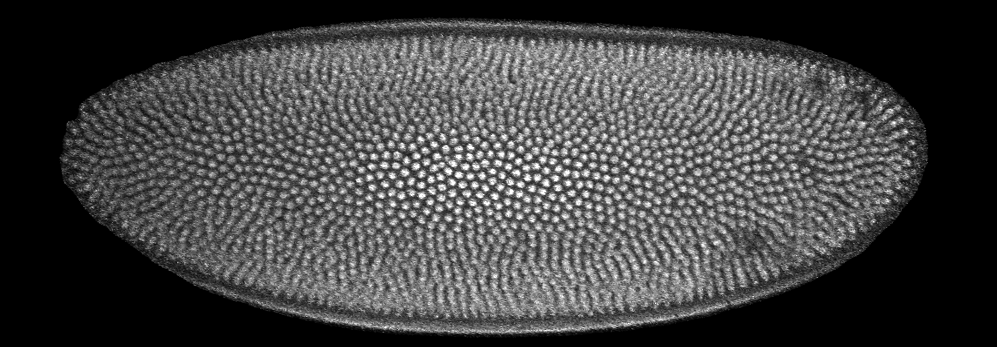

Supplement: Additional data file 1 — All TIFF files used to analyze nuclear movements in 22 living Histone2A-GFP embryos. [file gb-2006-7-12-r124-S1.zip › 171105e3/step-03.tif]

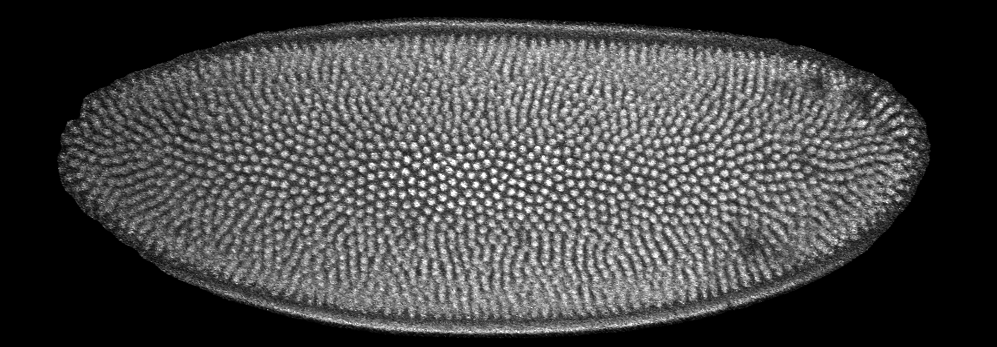

Supplement: Additional data file 1 — All TIFF files used to analyze nuclear movements in 22 living Histone2A-GFP embryos. [file gb-2006-7-12-r124-S1.zip › 171105e3/step-04.tif]

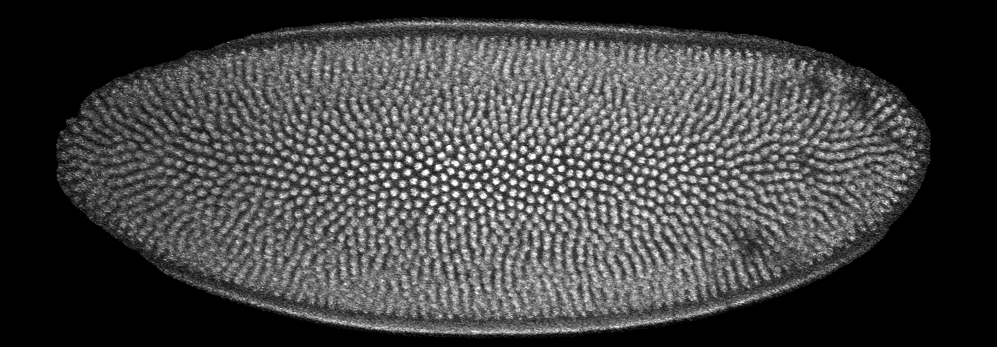

Supplement: Additional data file 1 — All TIFF files used to analyze nuclear movements in 22 living Histone2A-GFP embryos. [file gb-2006-7-12-r124-S1.zip › 171105e3/step-05.tif]

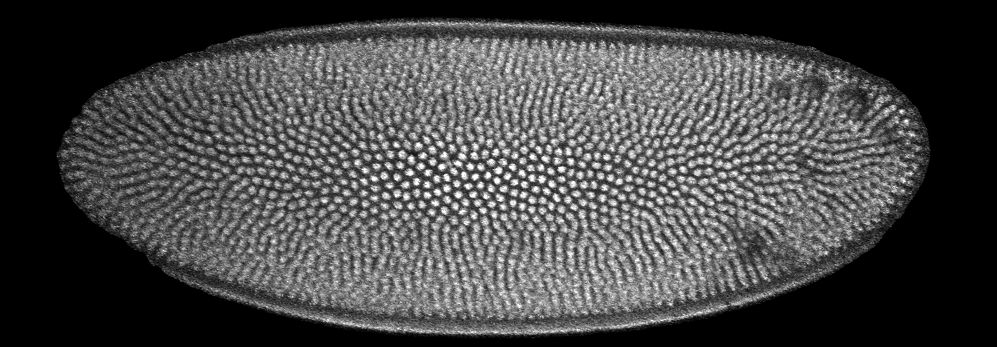

Supplement: Additional data file 1 — All TIFF files used to analyze nuclear movements in 22 living Histone2A-GFP embryos. [file gb-2006-7-12-r124-S1.zip › 171105e3/step-07.tif]

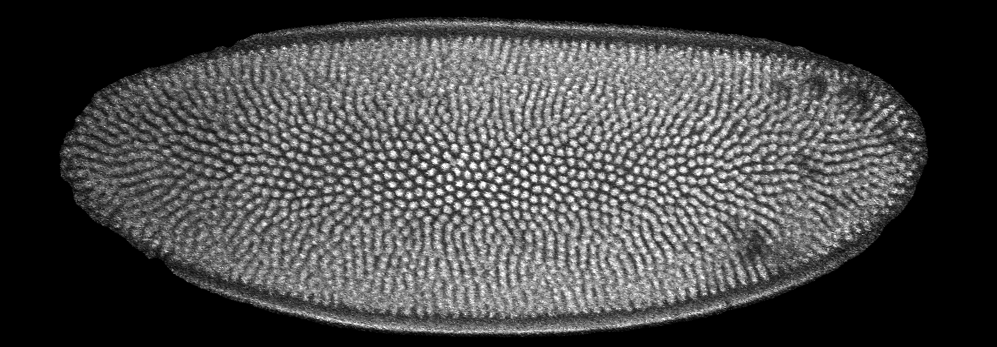

Supplement: Additional data file 1 — All TIFF files used to analyze nuclear movements in 22 living Histone2A-GFP embryos. [file gb-2006-7-12-r124-S1.zip › 171105e3/step-08.tif]

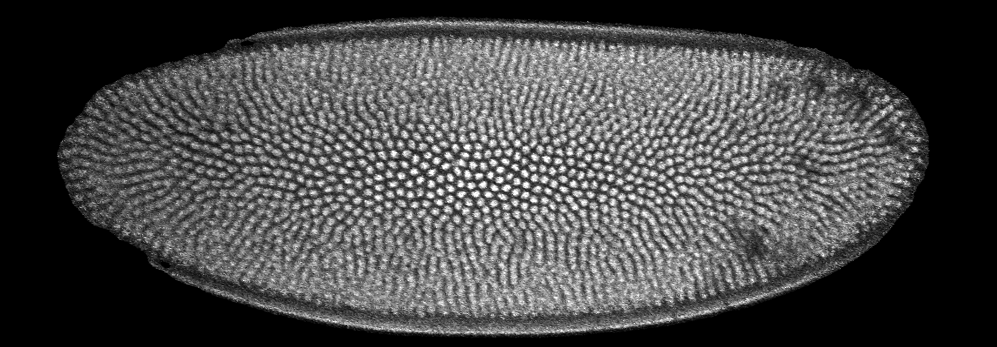

Supplement: Additional data file 1 — All TIFF files used to analyze nuclear movements in 22 living Histone2A-GFP embryos. [file gb-2006-7-12-r124-S1.zip › 171105e3/step-09.tif]

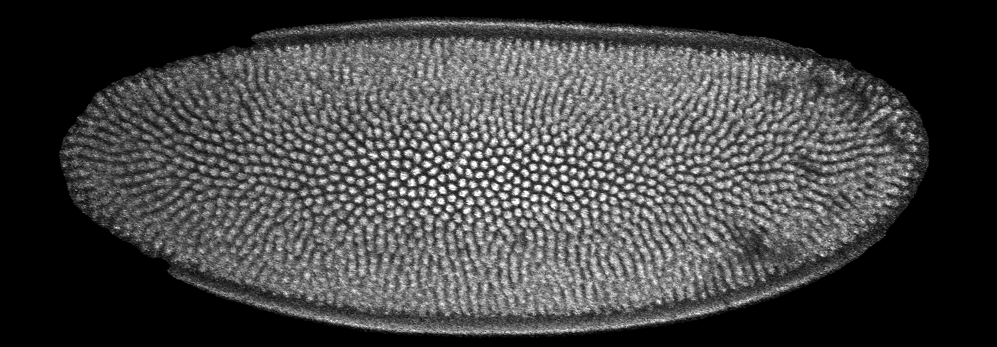

Supplement: Additional data file 1 — All TIFF files used to analyze nuclear movements in 22 living Histone2A-GFP embryos. [file gb-2006-7-12-r124-S1.zip › 171105e3/step-10.tif]

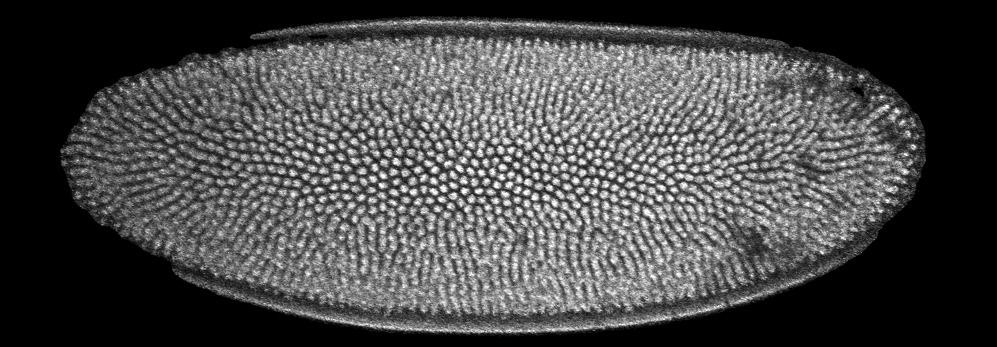

Supplement: Additional data file 1 — All TIFF files used to analyze nuclear movements in 22 living Histone2A-GFP embryos. [file gb-2006-7-12-r124-S1.zip › 171105e3/step-11.tif]

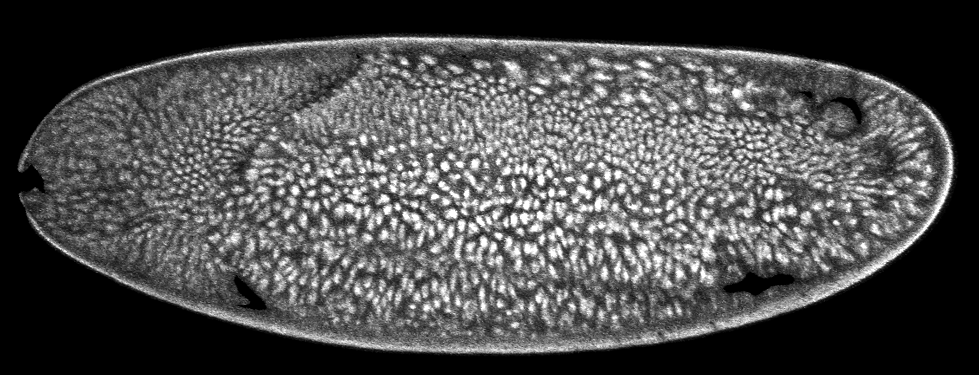

Supplement: Additional data file 1 — All TIFF files used to analyze nuclear movements in 22 living Histone2A-GFP embryos. [file gb-2006-7-12-r124-S1.zip › 171105e3/gastrulated.tif]

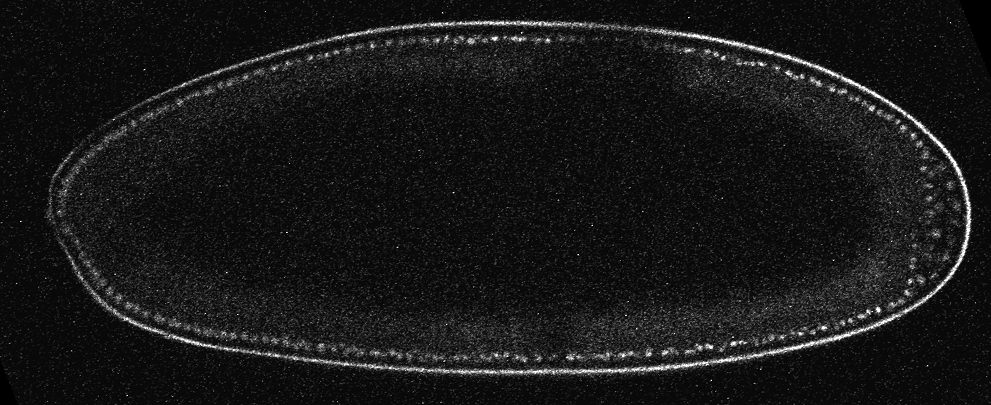

Supplement: Additional data file 1 — All TIFF files used to analyze nuclear movements in 22 living Histone2A-GFP embryos. [file gb-2006-7-12-r124-S1.zip › 171105e4/slice-0.tif]

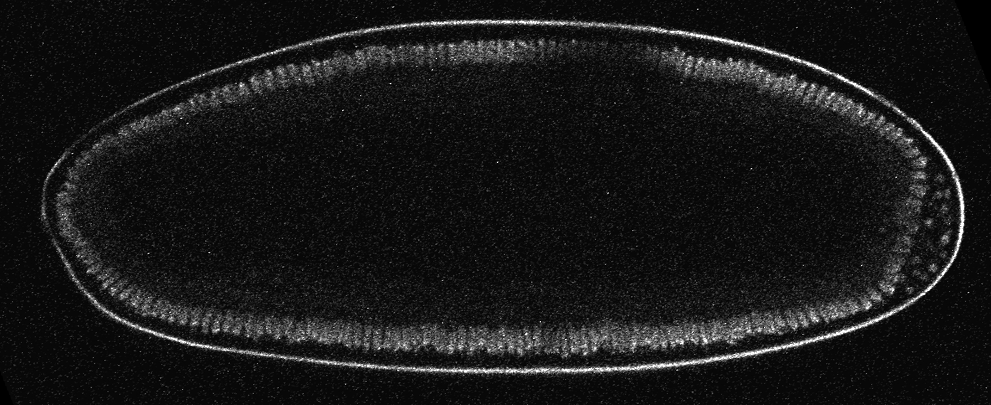

Supplement: Additional data file 1 — All TIFF files used to analyze nuclear movements in 22 living Histone2A-GFP embryos. [file gb-2006-7-12-r124-S1.zip › 171105e4/slice-1.tif]

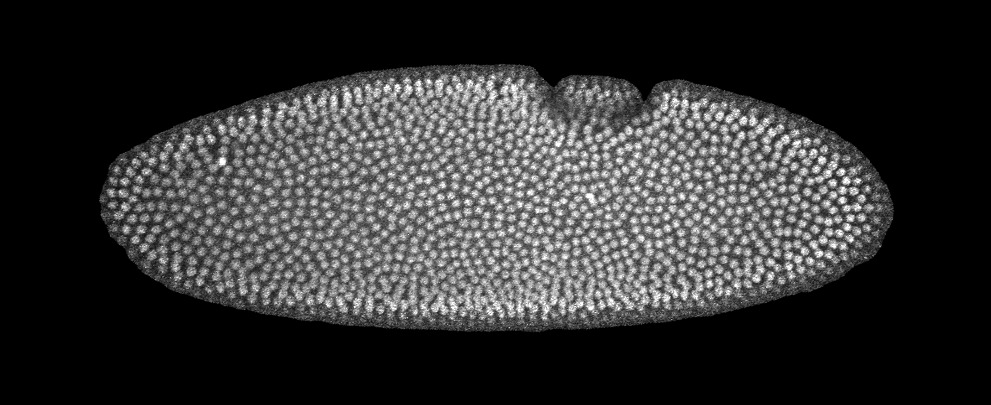

Supplement: Additional data file 1 — All TIFF files used to analyze nuclear movements in 22 living Histone2A-GFP embryos. [file gb-2006-7-12-r124-S1.zip › 171105e4/step-00.tif]

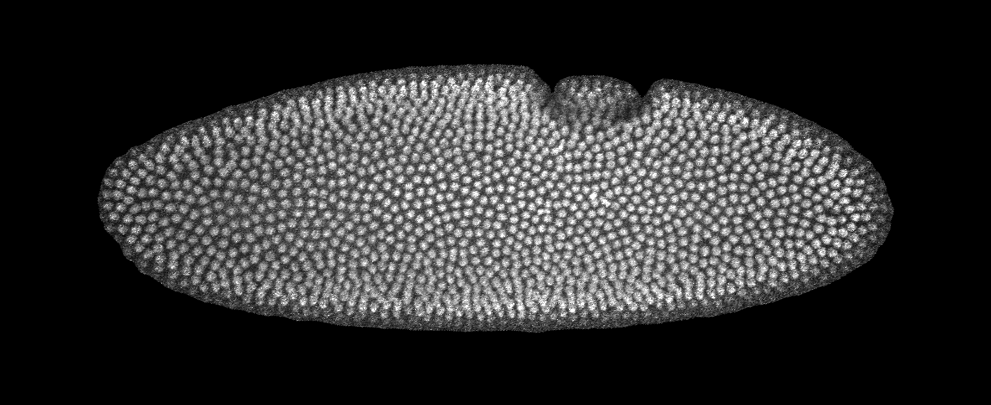

Supplement: Additional data file 1 — All TIFF files used to analyze nuclear movements in 22 living Histone2A-GFP embryos. [file gb-2006-7-12-r124-S1.zip › 171105e4/step-01.tif]

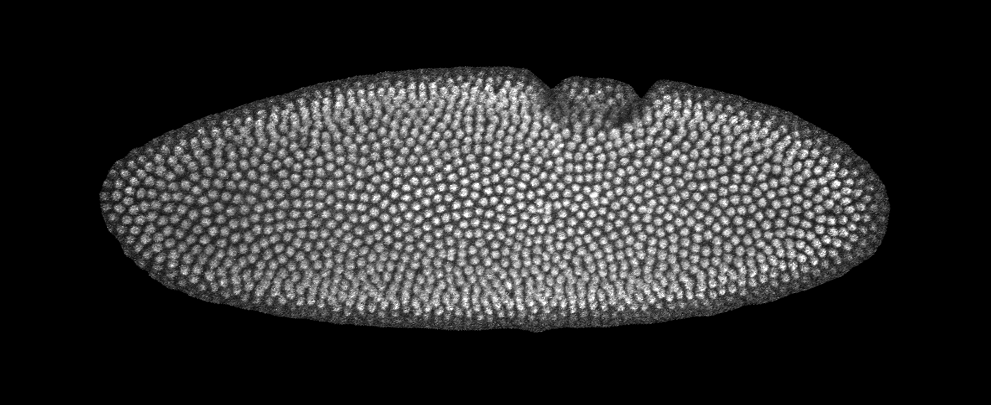

Supplement: Additional data file 1 — All TIFF files used to analyze nuclear movements in 22 living Histone2A-GFP embryos. [file gb-2006-7-12-r124-S1.zip › 171105e4/step-02.tif]

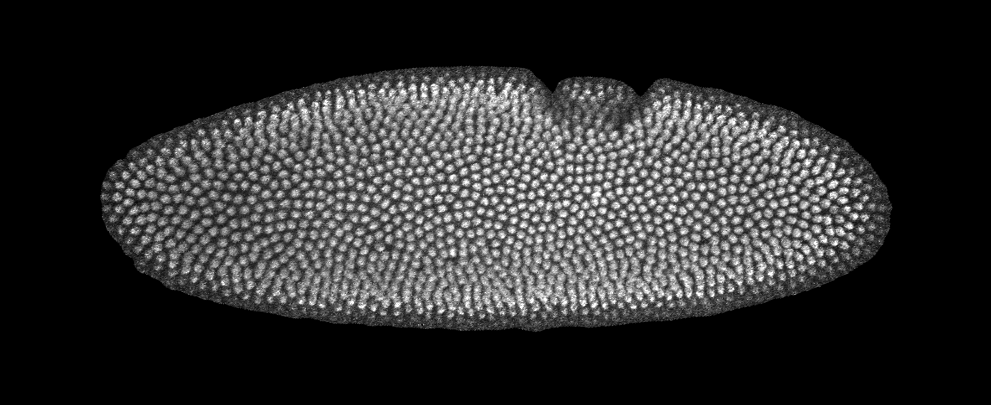

Supplement: Additional data file 1 — All TIFF files used to analyze nuclear movements in 22 living Histone2A-GFP embryos. [file gb-2006-7-12-r124-S1.zip › 171105e4/step-03.tif]

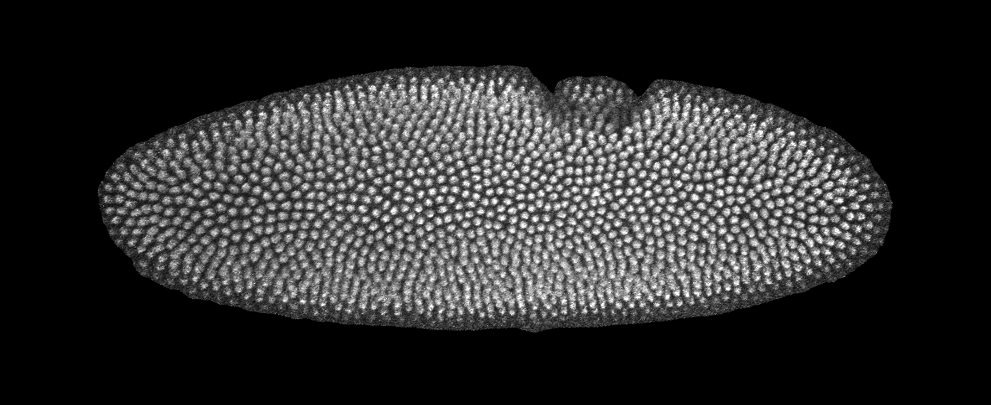

Supplement: Additional data file 1 — All TIFF files used to analyze nuclear movements in 22 living Histone2A-GFP embryos. [file gb-2006-7-12-r124-S1.zip › 171105e4/step-04.tif]

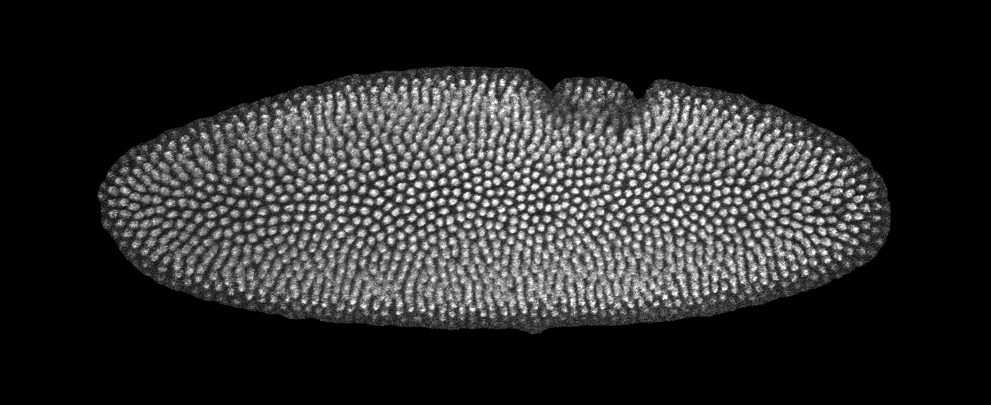

Supplement: Additional data file 1 — All TIFF files used to analyze nuclear movements in 22 living Histone2A-GFP embryos. [file gb-2006-7-12-r124-S1.zip › 171105e4/step-05.tif]

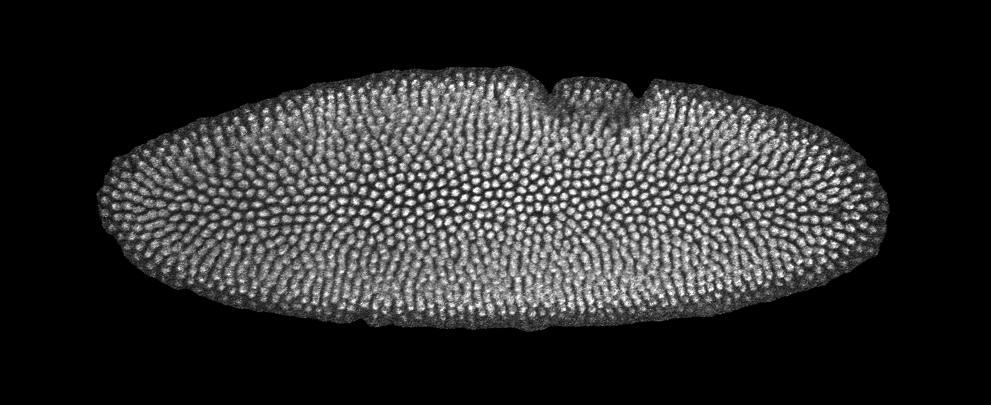

Supplement: Additional data file 1 — All TIFF files used to analyze nuclear movements in 22 living Histone2A-GFP embryos. [file gb-2006-7-12-r124-S1.zip › 171105e4/step-06.tif]

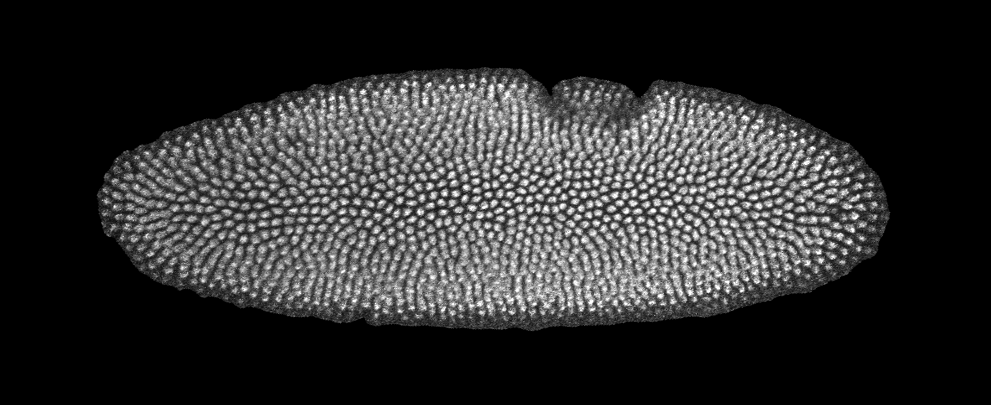

Supplement: Additional data file 1 — All TIFF files used to analyze nuclear movements in 22 living Histone2A-GFP embryos. [file gb-2006-7-12-r124-S1.zip › 171105e4/step-07.tif]

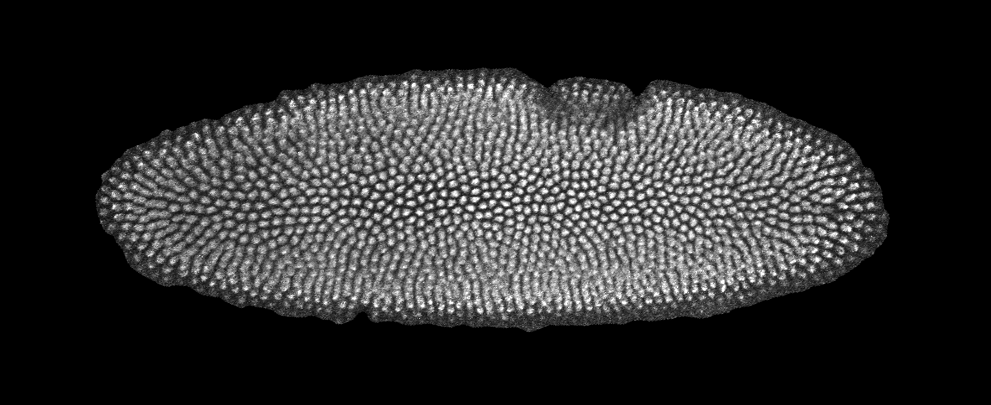

Supplement: Additional data file 1 — All TIFF files used to analyze nuclear movements in 22 living Histone2A-GFP embryos. [file gb-2006-7-12-r124-S1.zip › 171105e4/step-08.tif]

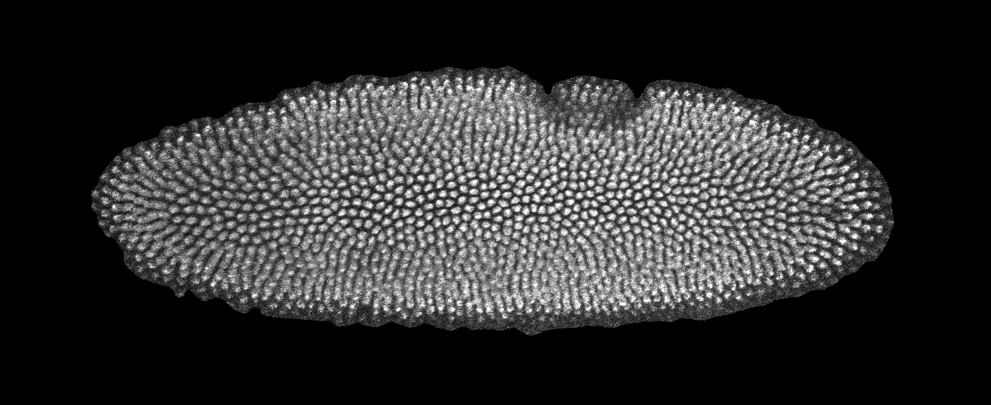

Supplement: Additional data file 1 — All TIFF files used to analyze nuclear movements in 22 living Histone2A-GFP embryos. [file gb-2006-7-12-r124-S1.zip › 171105e4/step-09.tif]

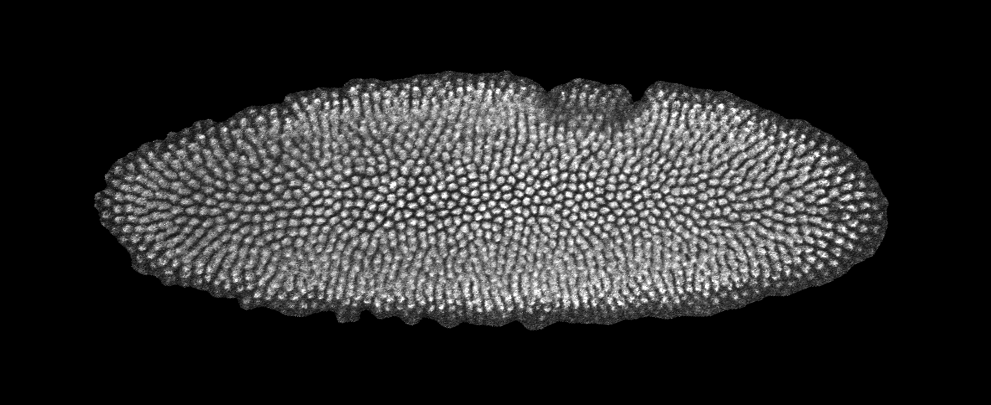

Supplement: Additional data file 1 — All TIFF files used to analyze nuclear movements in 22 living Histone2A-GFP embryos. [file gb-2006-7-12-r124-S1.zip › 171105e4/step-10.tif]

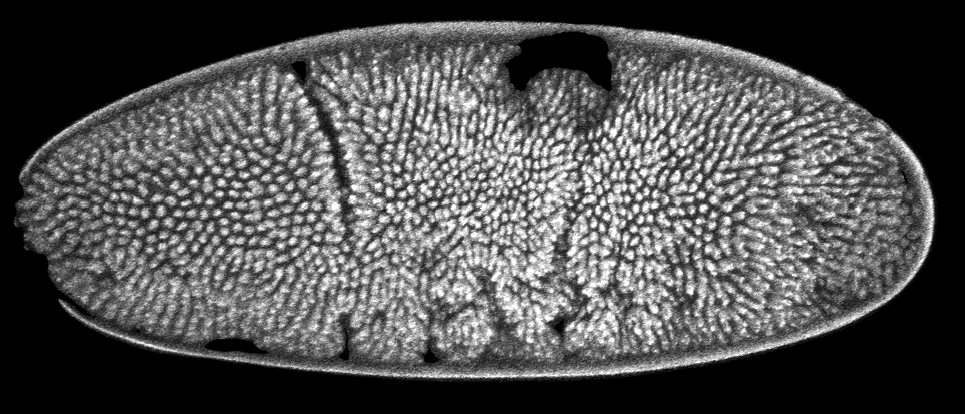

Supplement: Additional data file 1 — All TIFF files used to analyze nuclear movements in 22 living Histone2A-GFP embryos. [file gb-2006-7-12-r124-S1.zip › 171105e4/gastrulated.tif]

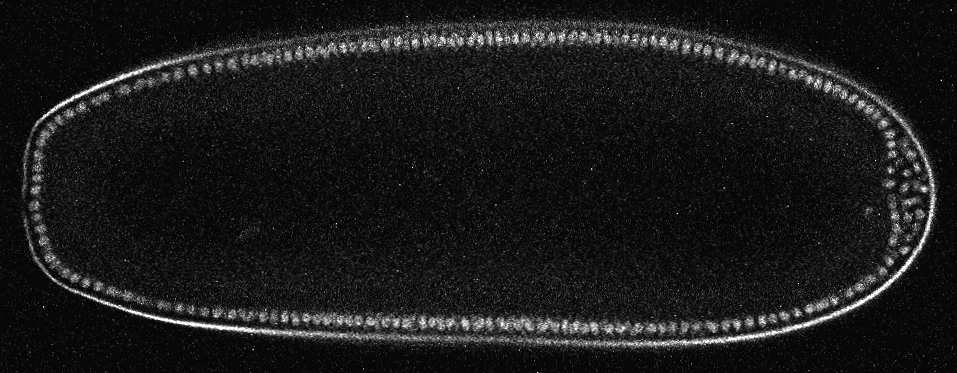

Supplement: Additional data file 1 — All TIFF files used to analyze nuclear movements in 22 living Histone2A-GFP embryos. [file gb-2006-7-12-r124-S1.zip › 191005e1/slice-0.tif]

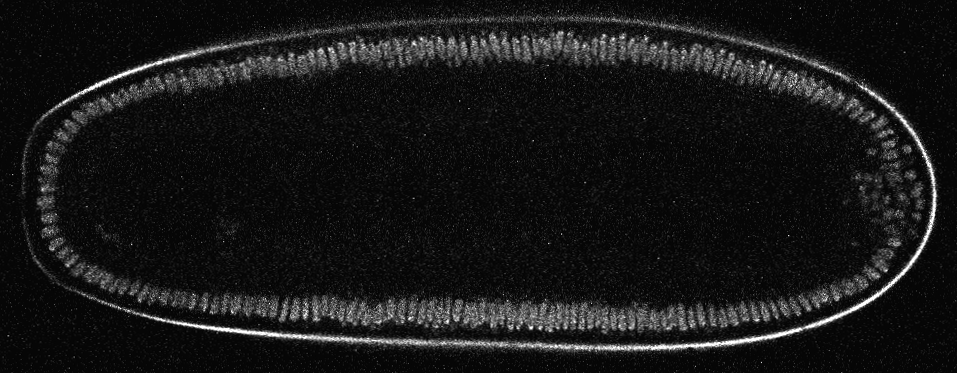

Supplement: Additional data file 1 — All TIFF files used to analyze nuclear movements in 22 living Histone2A-GFP embryos. [file gb-2006-7-12-r124-S1.zip › 191005e1/slice-1.tif]

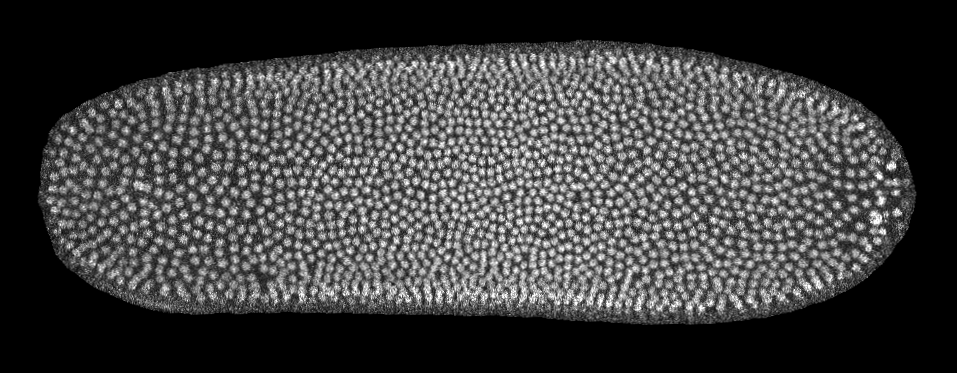

Supplement: Additional data file 1 — All TIFF files used to analyze nuclear movements in 22 living Histone2A-GFP embryos. [file gb-2006-7-12-r124-S1.zip › 191005e1/step-00.tif]

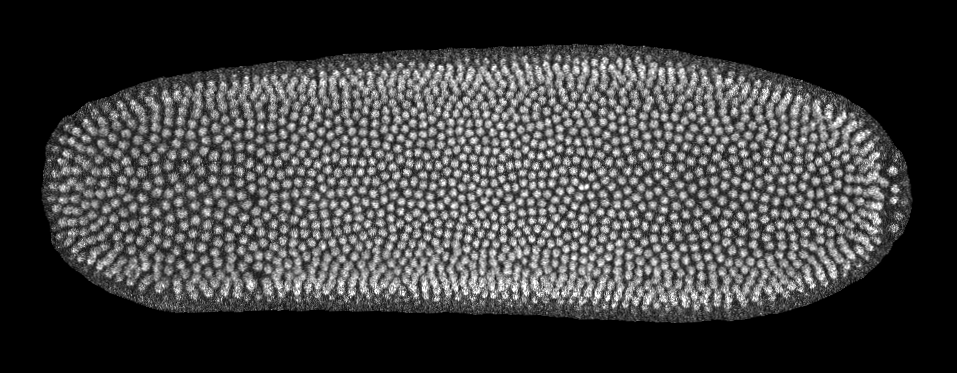

Supplement: Additional data file 1 — All TIFF files used to analyze nuclear movements in 22 living Histone2A-GFP embryos. [file gb-2006-7-12-r124-S1.zip › 191005e1/step-01.tif]

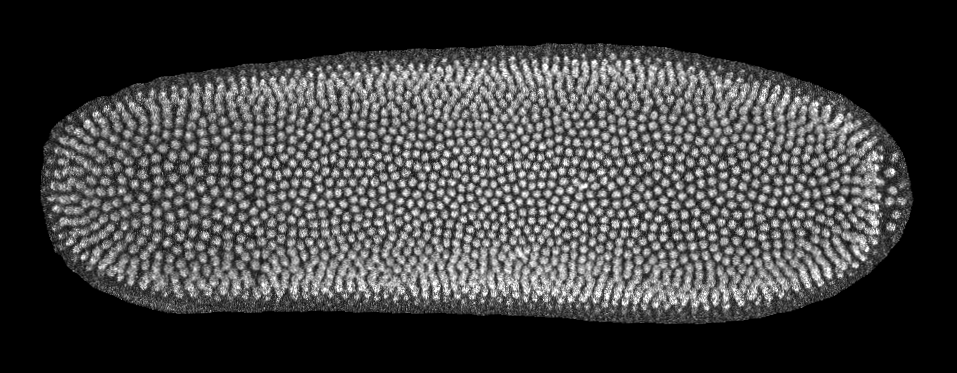

Supplement: Additional data file 1 — All TIFF files used to analyze nuclear movements in 22 living Histone2A-GFP embryos. [file gb-2006-7-12-r124-S1.zip › 191005e1/step-02.tif]

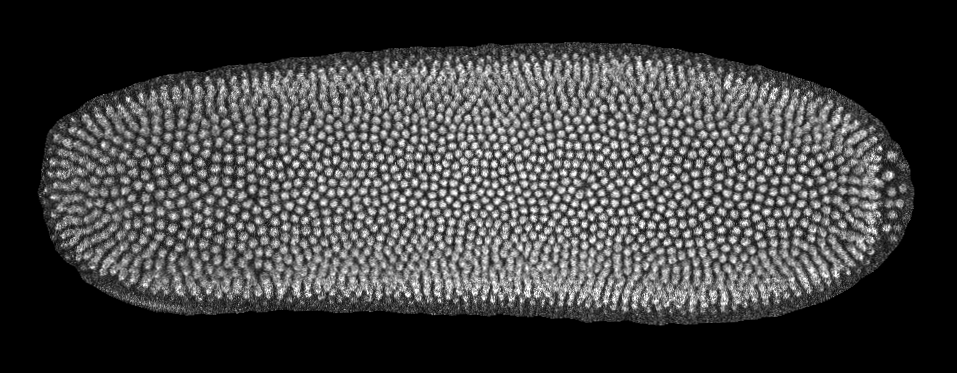

Supplement: Additional data file 1 — All TIFF files used to analyze nuclear movements in 22 living Histone2A-GFP embryos. [file gb-2006-7-12-r124-S1.zip › 191005e1/step-03.tif]

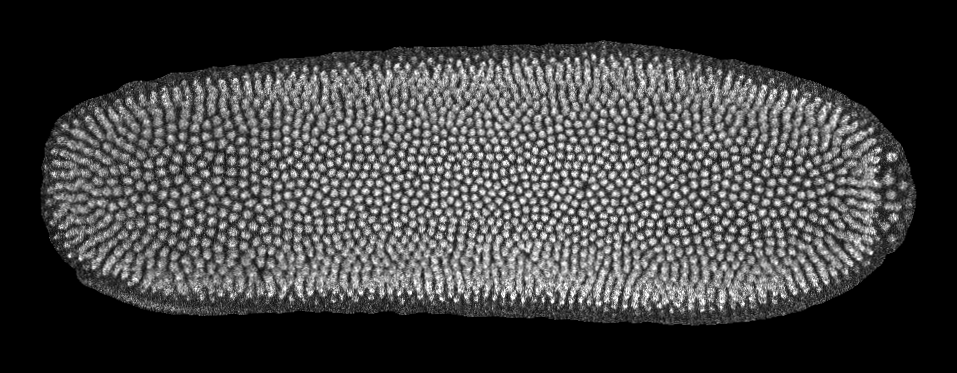

Supplement: Additional data file 1 — All TIFF files used to analyze nuclear movements in 22 living Histone2A-GFP embryos. [file gb-2006-7-12-r124-S1.zip › 191005e1/step-04.tif]

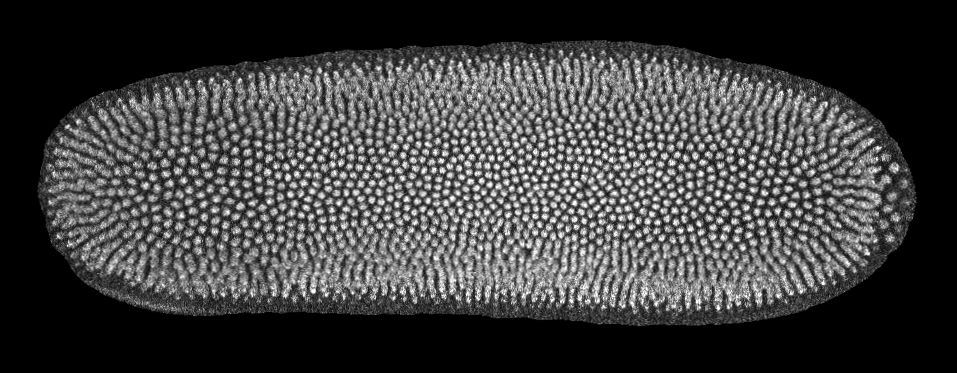

Supplement: Additional data file 1 — All TIFF files used to analyze nuclear movements in 22 living Histone2A-GFP embryos. [file gb-2006-7-12-r124-S1.zip › 191005e1/step-05.tif]

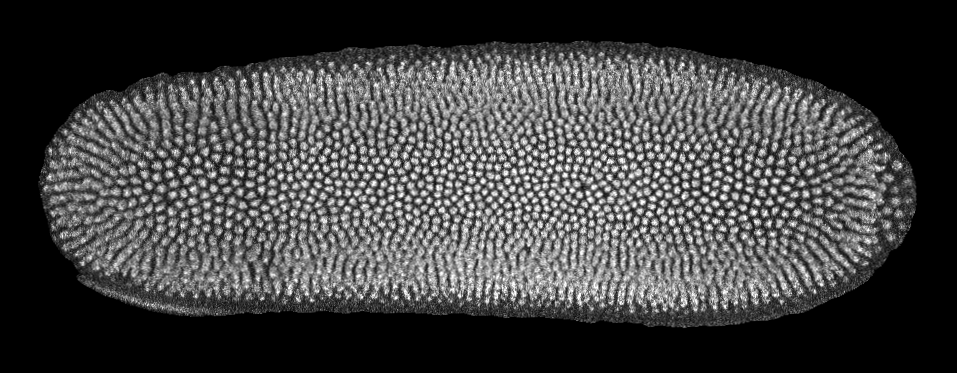

Supplement: Additional data file 1 — All TIFF files used to analyze nuclear movements in 22 living Histone2A-GFP embryos. [file gb-2006-7-12-r124-S1.zip › 191005e1/step-06.tif]

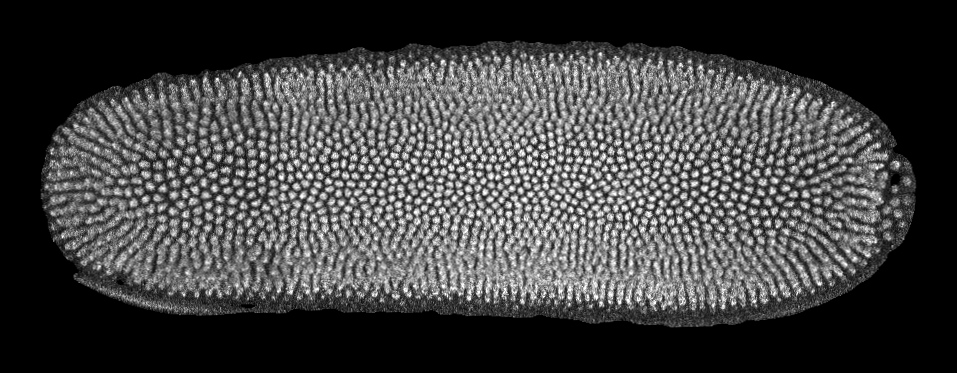

Supplement: Additional data file 1 — All TIFF files used to analyze nuclear movements in 22 living Histone2A-GFP embryos. [file gb-2006-7-12-r124-S1.zip › 191005e1/step-07.tif]

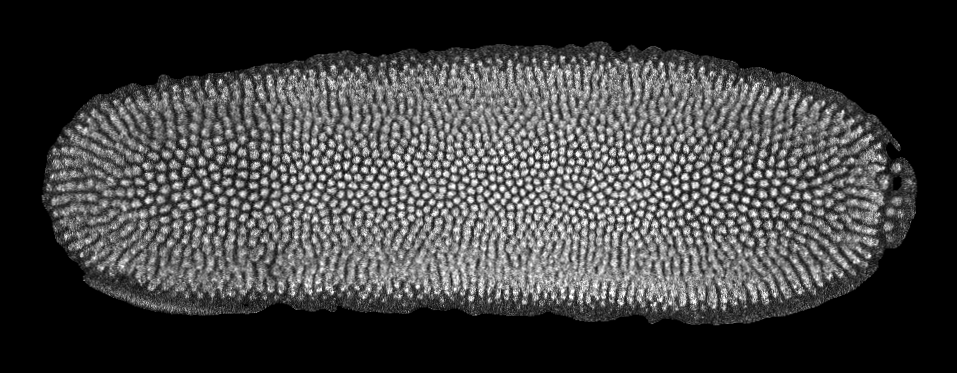

Supplement: Additional data file 1 — All TIFF files used to analyze nuclear movements in 22 living Histone2A-GFP embryos. [file gb-2006-7-12-r124-S1.zip › 191005e1/step-08.tif]

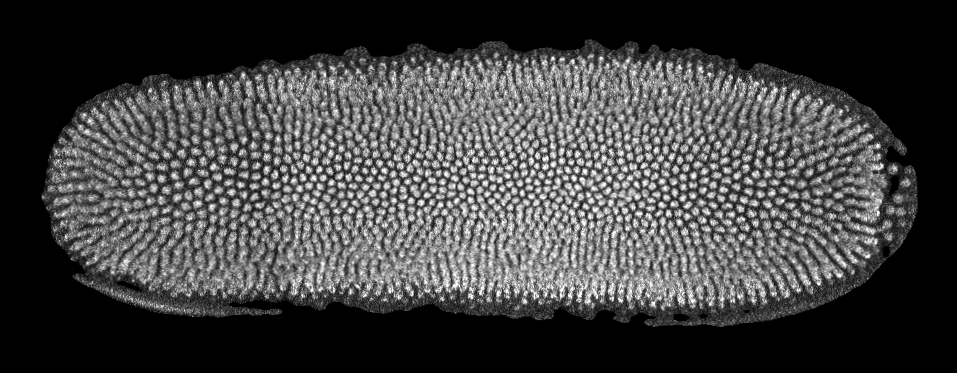

Supplement: Additional data file 1 — All TIFF files used to analyze nuclear movements in 22 living Histone2A-GFP embryos. [file gb-2006-7-12-r124-S1.zip › 191005e1/step-09.tif]

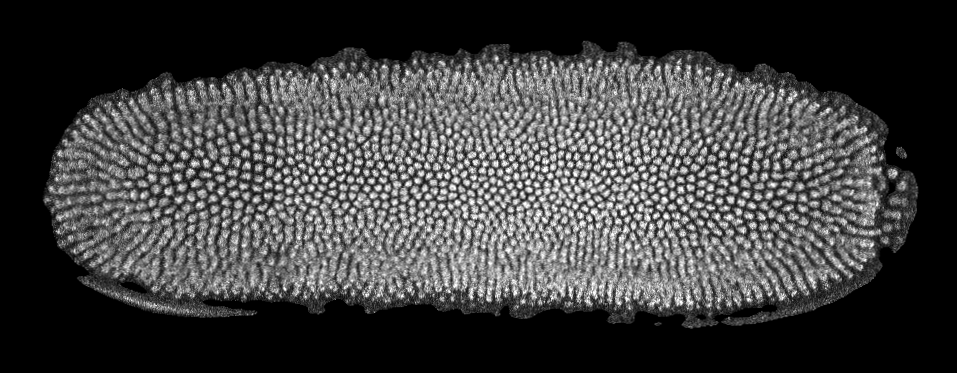

Supplement: Additional data file 1 — All TIFF files used to analyze nuclear movements in 22 living Histone2A-GFP embryos. [file gb-2006-7-12-r124-S1.zip › 191005e1/step-10.tif]

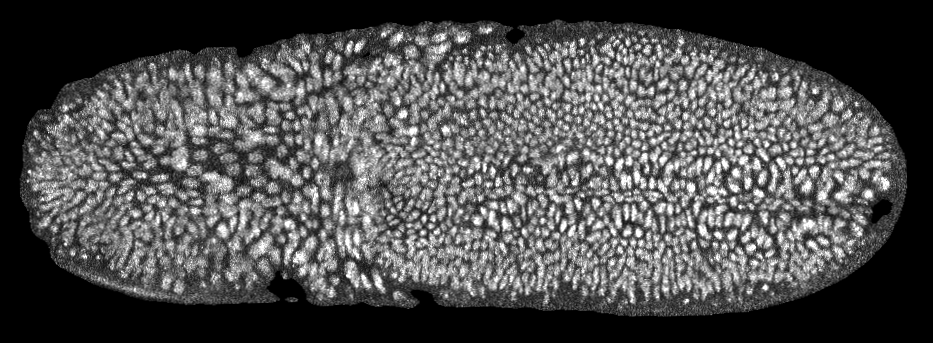

Supplement: Additional data file 1 — All TIFF files used to analyze nuclear movements in 22 living Histone2A-GFP embryos. [file gb-2006-7-12-r124-S1.zip › 191005e1/gastrulated.tif]

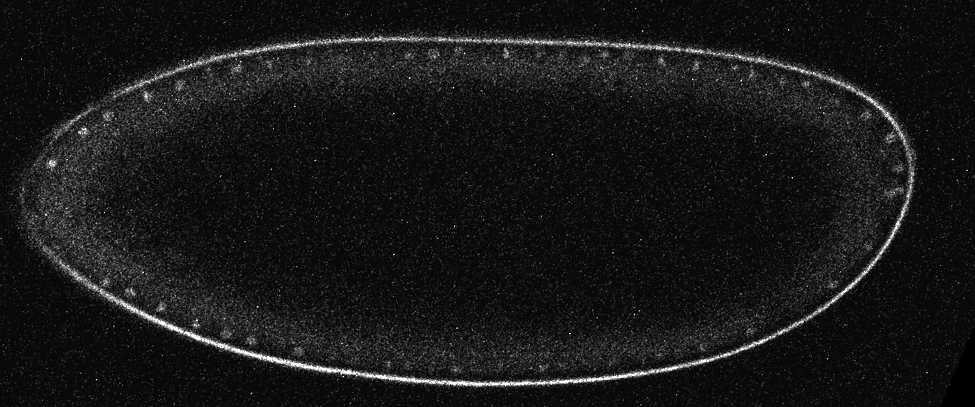

Supplement: Additional data file 1 — All TIFF files used to analyze nuclear movements in 22 living Histone2A-GFP embryos. [file gb-2006-7-12-r124-S1.zip › 191005e2/slice-0.tif]

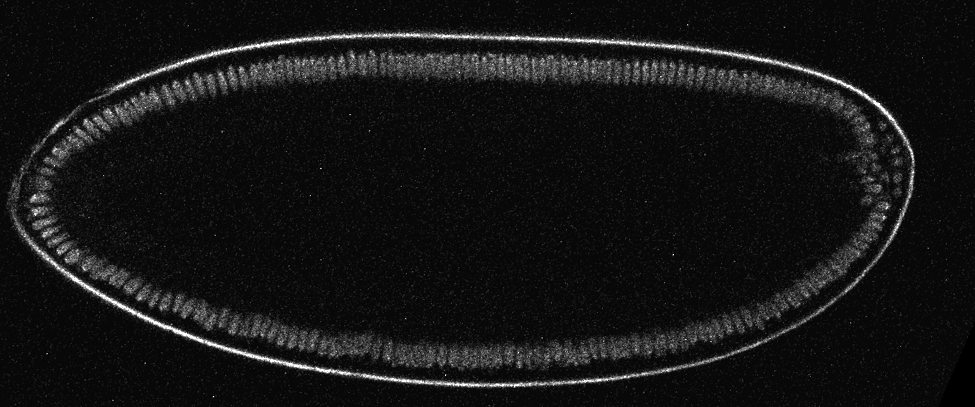

Supplement: Additional data file 1 — All TIFF files used to analyze nuclear movements in 22 living Histone2A-GFP embryos. [file gb-2006-7-12-r124-S1.zip › 191005e2/slice-1.tif]

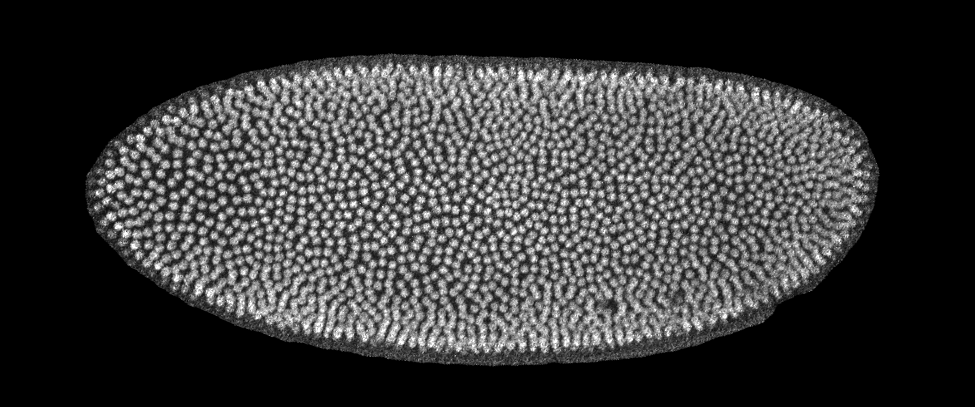

Supplement: Additional data file 1 — All TIFF files used to analyze nuclear movements in 22 living Histone2A-GFP embryos. [file gb-2006-7-12-r124-S1.zip › 191005e2/step-00.tif]

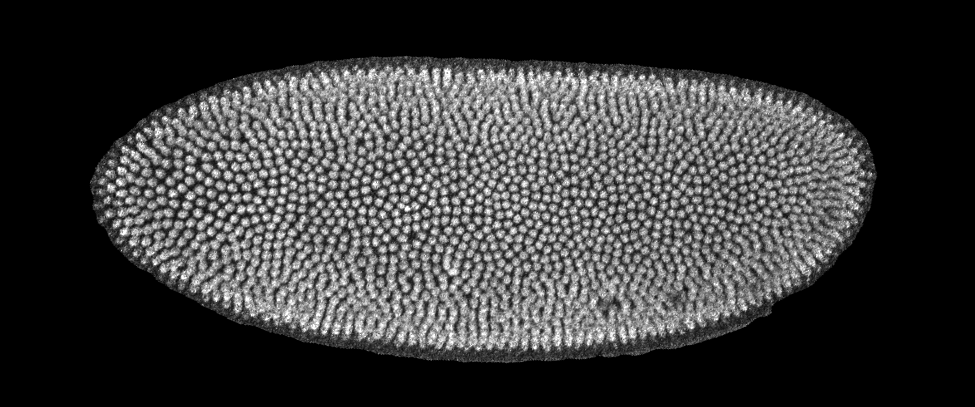

Supplement: Additional data file 1 — All TIFF files used to analyze nuclear movements in 22 living Histone2A-GFP embryos. [file gb-2006-7-12-r124-S1.zip › 191005e2/step-01.tif]

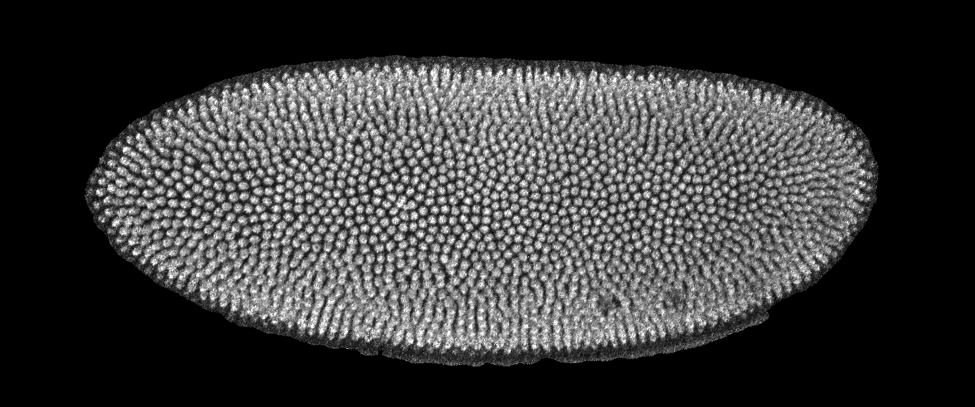

Supplement: Additional data file 1 — All TIFF files used to analyze nuclear movements in 22 living Histone2A-GFP embryos. [file gb-2006-7-12-r124-S1.zip › 191005e2/step-02.tif]

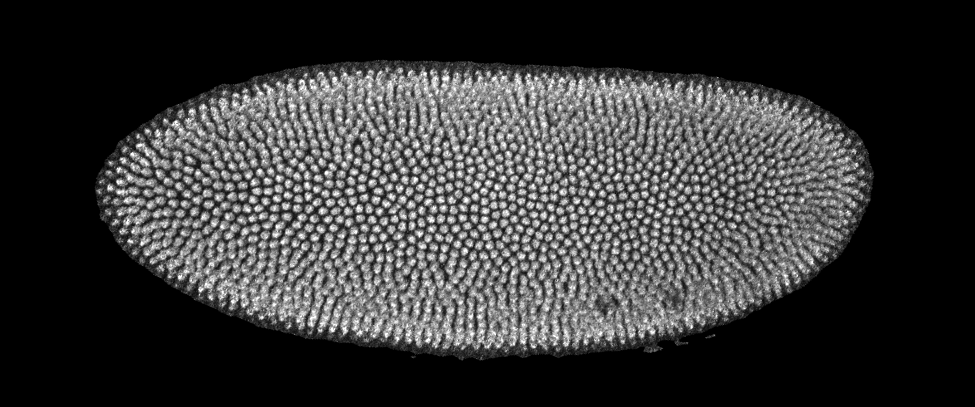

Supplement: Additional data file 1 — All TIFF files used to analyze nuclear movements in 22 living Histone2A-GFP embryos. [file gb-2006-7-12-r124-S1.zip › 191005e2/step-03.tif]

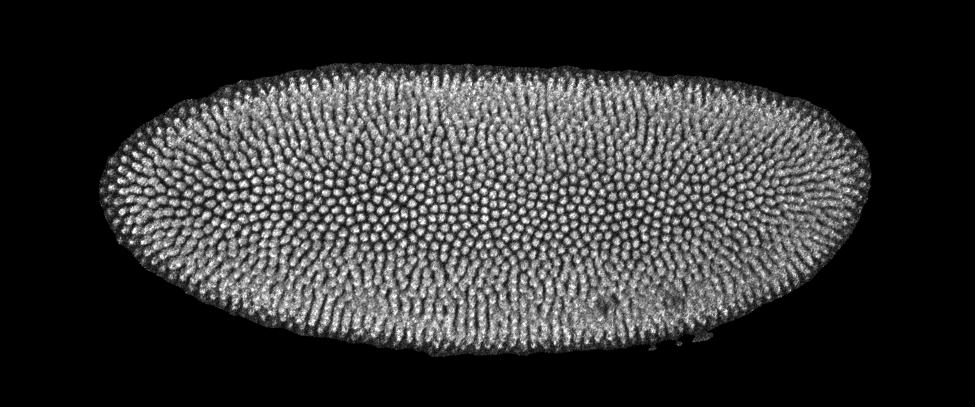

Supplement: Additional data file 1 — All TIFF files used to analyze nuclear movements in 22 living Histone2A-GFP embryos. [file gb-2006-7-12-r124-S1.zip › 191005e2/step-04.tif]

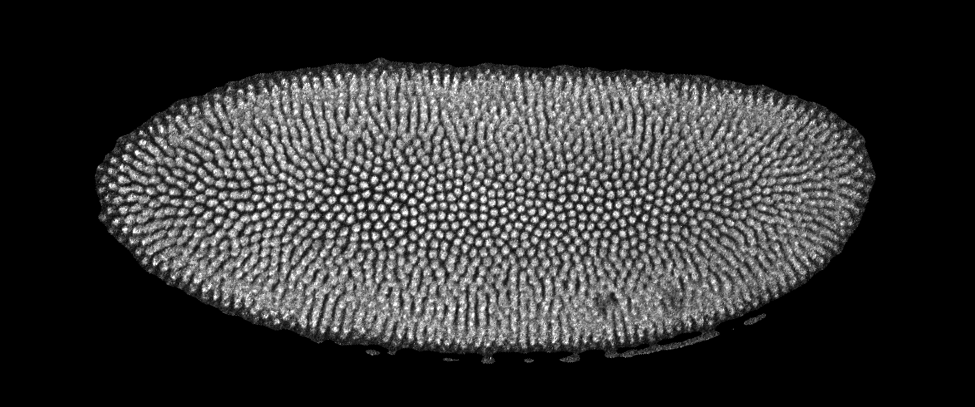

Supplement: Additional data file 1 — All TIFF files used to analyze nuclear movements in 22 living Histone2A-GFP embryos. [file gb-2006-7-12-r124-S1.zip › 191005e2/step-05.tif]

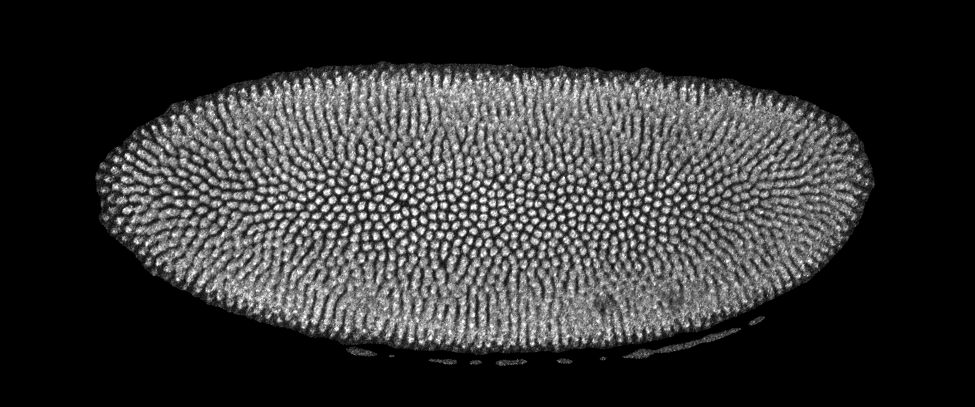

Supplement: Additional data file 1 — All TIFF files used to analyze nuclear movements in 22 living Histone2A-GFP embryos. [file gb-2006-7-12-r124-S1.zip › 191005e2/step-06.tif]

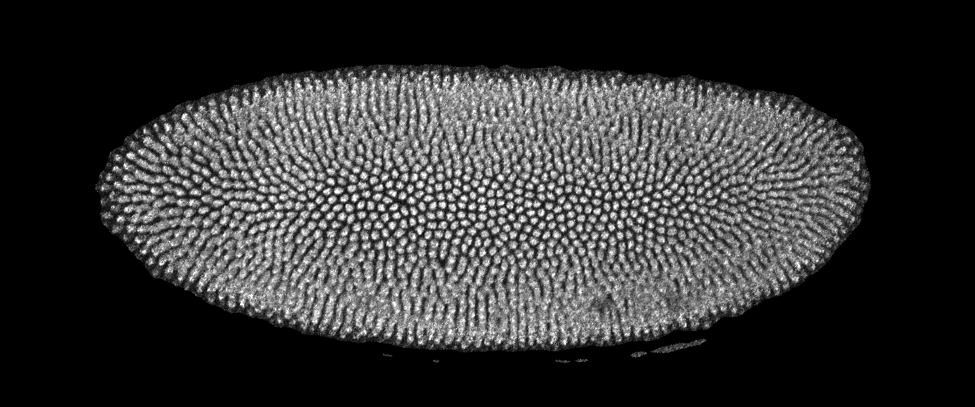

Supplement: Additional data file 1 — All TIFF files used to analyze nuclear movements in 22 living Histone2A-GFP embryos. [file gb-2006-7-12-r124-S1.zip › 191005e2/step-07.tif]

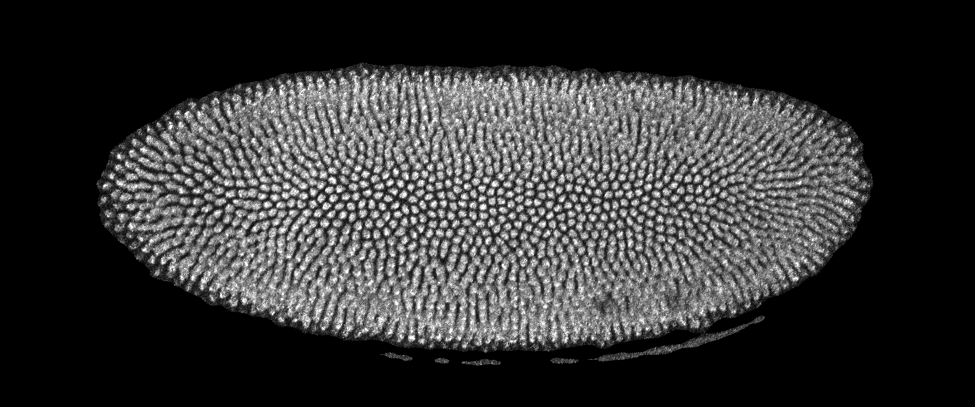

Supplement: Additional data file 1 — All TIFF files used to analyze nuclear movements in 22 living Histone2A-GFP embryos. [file gb-2006-7-12-r124-S1.zip › 191005e2/step-08.tif]

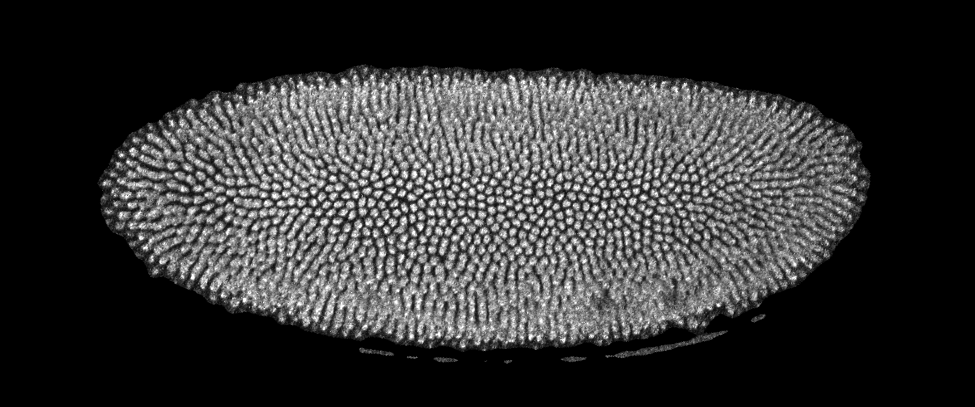

Supplement: Additional data file 1 — All TIFF files used to analyze nuclear movements in 22 living Histone2A-GFP embryos. [file gb-2006-7-12-r124-S1.zip › 191005e2/step-09.tif]

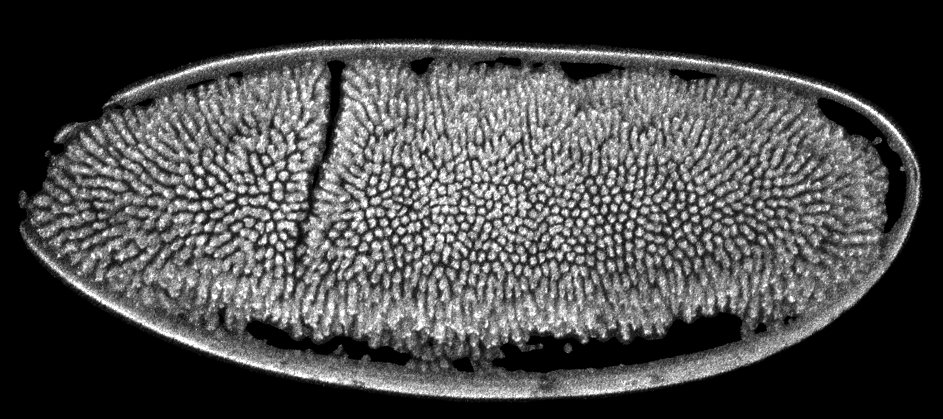

Supplement: Additional data file 1 — All TIFF files used to analyze nuclear movements in 22 living Histone2A-GFP embryos. [file gb-2006-7-12-r124-S1.zip › 191005e2/gastrulated.tif]
